# Splicing-Mediated Autoregulation Modulates Rpl22p Expression in *Saccharomyces cerevisiae*

**DOI:** 10.1371/journal.pgen.1005999

**Published:** 2016-04-20

**Authors:** Jason Gabunilas, Guillaume Chanfreau

**Affiliations:** 1 Department of Chemistry and Biochemistry, University of California, Los Angeles, Los Angeles, California, United States of America; 2 Molecular Biology Institute, University of California, Los Angeles, Los Angeles, California, United States of America; University of California San Francisco, UNITED STATES

## Abstract

In *Saccharomyces cerevisiae*, splicing is critical for expression of ribosomal protein genes (RPGs), which are among the most highly expressed genes and are tightly regulated according to growth and environmental conditions. However, knowledge of the precise mechanisms by which RPG pre-mRNA splicing is regulated on a gene-by-gene basis is lacking. Here we show that Rpl22p has an extraribosomal role in the inhibition of splicing of the *RPL22B* pre-mRNA transcript. A stem loop secondary structure within the intron is necessary for pre-mRNA binding by Rpl22p *in vivo* and splicing inhibition *in vivo* and *in vitro* and can rescue splicing inhibition *in vitro* when added in *trans* to splicing reactions. Splicing inhibition by Rpl22p may be partly attributed to the reduction of co-transcriptional U1 snRNP recruitment to the pre-mRNA at the *RPL22B* locus. We further demonstrate that the inhibition of *RPL22B* pre-mRNA splicing contributes to the down-regulation of mature transcript during specific stress conditions, and provide evidence hinting at a regulatory role for this mechanism in conditions of suppressed ribosome biogenesis. These results demonstrate an autoregulatory mechanism that fine-tunes the expression of the Rpl22 protein and by extension Rpl22p paralog composition according to the cellular demands for ribosome biogenesis.

## Author Summary

Ribosomes are responsible for protein production in all living cells, serving as the grounds for the translation of genetic information from RNA to protein. Given the vital role of the ribosome in protein assembly, ribosome components are highly expressed and are subject to tight regulation. Some ribosomal proteins are also known to engage in extra-ribosomal activities. In our study, we demonstrate that the ribosomal protein Rpl22p is able to regulate its own expression by inhibiting the processing of its own RNA transcript, leading to degradation of the RNA. We also show that this self-imposed regulation plays a role in limiting *RPL22* transcript levels in specific stress conditions. We suggest that this mechanism may impact the composition of ribosomes by influencing the availability of the Rpl22p paralogs.

## Introduction

Ribosomal protein genes (RPGs) constitute a majority of the most frequently transcribed genes in the budding yeast *Saccharomyces cerevisiae* [1]. Due in part to their high levels of expression and their vital role as components of the translational machinery, understanding of the regulation of RPG expression has garnered considerable attention. While RPGs are tightly regulated at the transcriptional level [2], the fact that nearly half of all intron-containing genes in *S*. *cerevisiae* are RPGs [3] has led to questions regarding the importance of these introns in RPG regulation. To address this, a comprehensive deletion of the yeast RPG intronome revealed numerous cases of intron-dependent intergenic and intragenic regulation of RPG expression that also impacted cell growth in various stress conditions [4]. These findings led to the conclusion that introns within RPGs govern the auto- and cross-regulation of RPG expression. While some structural elements within intronic RPGs were found to be important for splicing efficiency [5,6], the precise mechanisms by which this regulation is achieved on a gene-by-gene basis remain largely unknown.

Over the past several decades, a few studies have shown that the regulation of expression of particular RPGs is in part dependent upon extra-ribosomal autoregulatory functions of the ribosomal proteins themselves [7]. In *S*. *cerevisiae*, some ribosomal proteins have been found to regulate the splicing of their own pre-mRNA transcripts. Such direct regulation has been characterized for the ribosomal protein Rpl30p, which binds to an RNA secondary structure in the precursor transcript and inhibits its splicing [8]. Similarly, the ribosomal protein Rps14p self-regulates its expression by directly binding to a stem loop structure in the *RPS14B* pre-mRNA [9], while the ribosomal protein Rps9p preferentially represses splicing of the *RPS9A* minor paralog through the recognition of an intronic structural element [10]. Other ribosomal proteins have been found to regulate their mRNAs by mechanisms other than splicing, particularly in cases where the nascent transcript does not contain an annotated intron. For instance, recent studies showed that yeast Rps28p indirectly binds a regulatory element in the 3’ untranslated region (3’UTR) of its mRNA transcript via Edc3p and targets the mRNA for decapping and degradation [11], while Rpl9p influences the transcription termination pathway of the *RPL9B* transcript, coupling termination to nuclear degradation [12]. RPG autoregulation is not limited to *S*.*cerevisiae* but has also been identified in higher eukaryotes. In mice and zebrafish the ribosomal protein Rpl22 regulates the expression of its paralog protein Rpl22l1 by interacting with the Rpl22l1 pre-mRNA, thereby repressing expression of the protein through an as-yet unknown mechanism [13].

We previously showed that the pre-mRNA of *RPL22B* contains an intronic alternative 5’ splice site and that splicing at this site gives rise to a transcript that is degraded by the cytoplasmic nonsense-mediated decay (NMD) pathway [14]. This finding suggested that alternative splicing of this precursor transcript may serve as a means for regulating mature *RPL22* transcript levels in an NMD-dependent manner. In this study, we describe an autoregulatory circuit for the regulation of *RPL22* in *S*.*cerevisiae* based on the inhibition of the splicing of the *RPL22B* pre-mRNA by Rpl22p. We identify and characterize an RNA stem loop within the *RPL22B* intron that is necessary for the inhibition of pre-mRNA splicing by Rpl22p *in vivo* and *in vitro*. Physiologically, we found that this mechanism promotes the down-regulation of spliced *RPL22B* transcript during stress. Together with our previous findings, these results demonstrate that *RPL22B* is precisely regulated at the RNA level by multiple splicing-based mechanisms and identify a physiological extraribosomal function of Rpl22p during stress.

## Results

### Splicing of the *RPL22B* pre-mRNA is regulated by the Rpl22p protein

For several duplicated genes in *S*. *cerevisiae*, including *RPL22*, deletion of one paralog results in a compensatory enhancement of expression of the remaining paralog [15,16,17]. However, the mechanisms by which this upregulation occurs are not always clear. Because the vast majority (>90%) of ribosomes contain Rpl22p produced from the *RPL22A* locus in wild-type yeast cells [18], we hypothesized that the loss of this gene may trigger a compensatory response with regards to expression and/or processing of the paralogous *RPL22B* transcript.

**Fig 1:**
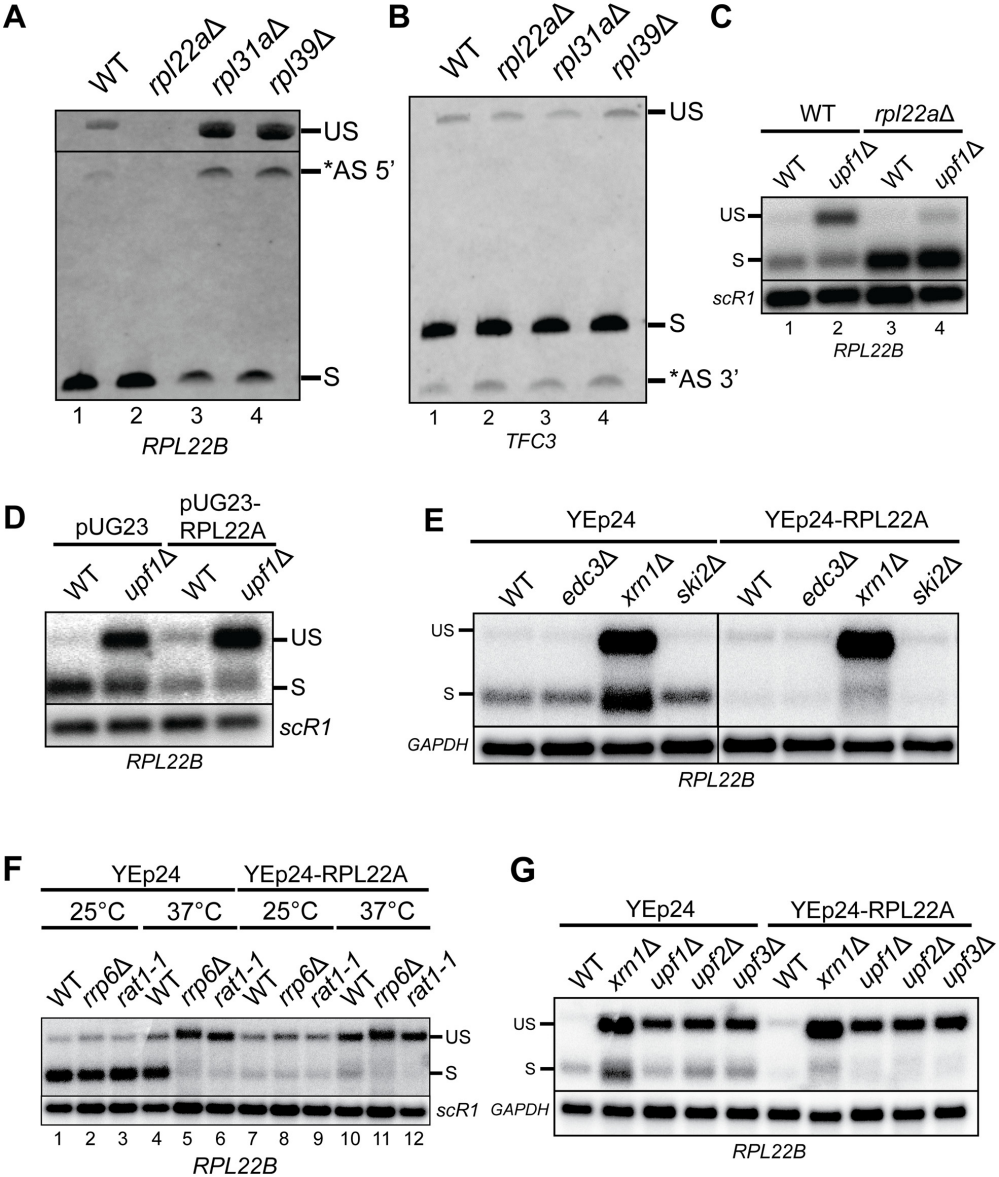
Rpl22Ap modulates the splicing of *RPL22B* pre-mRNA, which is targeted for degradation by cytoplasmic and nuclear decay factors. **A.** RT-PCR analysis of splicing products of the *RPL22B* pre-mRNA in wild-type and ribosomal protein deletion mutants. Bands indicate the unspliced (US), alternatively-spliced (*AS 5’), and spliced (S) species. **B.** RT-PCR analysis of splicing products of the *TFC3* pre-mRNA in wild-type and ribosomal protein deletion mutants. Bands indicate the unspliced (US), spliced (S), and alternatively spliced (*AS 3’) species. **C.** Northern blot analysis of *RPL22B* splicing products in wild-type, *upf1*Δ, *rpl22a*Δ, and *upf1*Δ/*rpl22a*Δ cells detected using an *RPL22B* 5’UTR riboprobe. Shown are the unspliced (US) and spliced (S) species. *SCR1* was used as a loading control. **D.** Northern blot showing *RPL22B* splicing products in wild-type or *upf1*Δ cells with an empty pUG23 vector or intronless *RPL22A* cDNA overexpression plasmid. Splicing products were detected using an *RPL22B* 5’UTR riboprobe. Labeling of the transcripts is similar to panel C. *SCR1* was used as a loading control. **E.** Northern blot analysis of *RPL22B* in cytoplasmic RNA decay mutants carrying the multi-copy YEp24 vector with either no insert or expressing the intronless *RPL22A* gene. Labeling of the transcripts is similar to panels C-D. *GAPDH* was used as a loading control. **F.** Northern blot analysis of *RPL22B* in nuclear RNA decay mutants carrying an empty YEp24 vector or an intronless *RPL22A* overexpression plasmid. Strains were grown to exponential phase in–URA media at 25°C and then shifted to 37°C for three hours. Labeling of the transcripts is similar to panels C-E. *SCR1* was used as a loading control. **G.** Northern blot analysis of *RPL22B* in *xrn1*Δ and NMD mutant strains carrying an empty YEp24 vector or an *RPL22A* overexpression plasmid. Detection methods and labeling are similar to panels C-F. *GAPDH* was used as a loading control.

To determine whether Rpl22p-mediated splicing regulation occurs for *RPL22B*, we performed RT-PCR using locus-specific *RPL22B* primers on cDNA derived from total RNA of wild-type cells and cells harboring the deletion of the *RPL22A* gene (*rpl22a*Δ). The deletion of *RPL22A* resulted in a substantial increase in the proportion of spliced transcript relative to the unspliced pre-mRNA and the 5’ alternatively spliced *RPL22B* transcript that is subject to NMD (Fig 1A). Because of the severe growth defect of the *rpl22a*Δ strain [17], we could not rule out that this splicing pattern was an indirect effect of slow growth, as growth impairment has been shown to confer multiple phenotypes in yeast including resistance to heat shock and ER stress [17,19]. Therefore, we also analyzed *RPL22B* splicing patterns in two other slow-growing RP mutants, *rpl31a*Δ and *rpl39*Δ ([20], kindly provided by B. Kennedy). We found that the splicing pattern for *RPL22B* was remarkably different in these two mutants in comparison to wildtype and *rpl22a*Δ. Specifically, these mutants both displayed an accumulation of the unspliced and alternative spliced transcripts while the proportion of spliced transcript was concomitantly reduced (Fig 1A). Therefore, the splicing pattern for *RPL22B* in the *rpl22a*Δ strain appears to be unique to that strain and does not result from a severe growth defect. To ensure that the reduction in pre-mRNA levels detected in the *rpl22a*Δ strain was specific to *RPL22B*, we repeated the RT-PCR analyses on each of these strains for *TFC3*, which contains an alternative 3’ splice site [14]. We found no observable differences in *TFC3* splicing patterns across these four strains (Fig 1B) in spite of considerable differences in generation time [20]. This result suggests that the splicing pattern for *RPL22B* in the *rpl22a*Δ mutant is unique to that strain and that the deletion of *RPL22A* does not confer a general change in splicing patterns. Interestingly, the levels of the 5’ alternatively spliced species did not increase in the *rpl22a*Δ strain but was instead decreased (Fig 1A, lane 2), suggesting that the inhibition of splicing is specific to the annotated splice site.

The precursor and alternatively spliced transcripts of *RPL22B* are degraded by NMD [14,21], making their accumulation difficult to detect in the context of an active NMD system. To better characterize the impact of *RPL22A* deletion on the splicing of *RPL22B*, we analyzed *RPL22B* levels by Northern blot in both wild-type and NMD-deficient (*upf1*Δ) strains in the presence or absence of *RPL22A*. As expected, disabling NMD led to the accumulation of the NMD-sensitive unspliced transcript while the deletion of *RPL22A* reduced the unspliced signal to undetectable levels (Fig 1C), in accordance with the results from the RT-PCR. Furthermore, the deletion of *RPL22A* attenuated the accumulation of the unspliced transcript in the *upf1*Δ mutant (Fig 1C, compare lanes 2 and 4), either because the degradation of this transcript is hyperactivated in the *rpl22a*Δ mutant or, alternatively, because the absence of *RPL22A* allows for more efficient splicing of the pre-mRNA. The latter hypothesis was supported by the observation that the *rpl22a*Δ deletion also resulted in a significant increase in the levels of spliced *RPL22B* transcript compared to wildtype (Fig 1C, lanes 1 and 3), a difference that was not detected by RT-PCR in Fig 1A, likely because of saturated amplification of the more abundant spliced cDNA in both samples.

Because the deletion of *RPL22A* enhanced the levels of spliced *RPL22B* transcript and repressed the levels of unspliced transcript, we reasoned that overexpressing Rpl22p would have the opposite effect and repress the levels of spliced transcript. To test this hypothesis, we created an *RPL22A* cDNA overexpression vector in which an intronless *RPL22A* cDNA was cloned into the pUG23 plasmid and placed under the control of the *MET25* promoter such that yeast cells transformed with this plasmid overexpress the *RPL22A* cDNA when grown in media lacking methionine [22]. Indeed, overexpression of the *RPL22A* cDNA resulted in the repression of levels of spliced *RPL22B* transcript and accumulation of the pre-mRNA (Fig 1D lanes 1 and 3). Notably, the accumulation of the pre-mRNA detected in the *upf1*Δ strain was even more pronounced when *RPL22A* was overexpressed (Fig 1D, lanes 2 and 4). We note that the impact of *RPL22A* overexpression on the *RPL22B* transcript splicing was not as dramatic as the effect of the *RPL22A* deletion, likely because the overexpression of an already highly-expressed gene such as *RPL22A* induces a minor perturbation compared to the deletion of the gene. Altogether, these results suggest that the splicing efficiency of the *RPL22B* transcript is heavily influenced by the levels of Rpl22p protein, the majority of which is derived from the *RPL22A* locus.

### Nuclear and cytoplasmic decay of the *RPL22B* pre-mRNA is not influenced by Rpl22p autoregulation

To determine whether other mechanisms contribute to the degradation of the unspliced *RPL22B* transcript, we analyzed the accumulation of the *RPL22B* pre-mRNA in deletion mutants for the cytoplasmic RNA decay factors Xrn1p [23] and the Ski2p activator of the exosome [24], as well as the mRNA decapping factor Edc3p which is involved in autoregulation of *RPS28B* [11]. We analyzed *RPL22B* pre-mRNA levels in these deletion mutants with or without the overexpression of intronless *RPL22A* from a multicopy YEp24 plasmid to determine whether the inhibition of splicing impacts the route of degradation taken by the pre-mRNA.

As expected, inactivation of Xrn1p resulted in an increase of both unspliced and spliced *RPL22B* transcript (Fig 1E), consistent with the general role of Xrn1p in cytoplasmic mRNA degradation [25]. By contrast, the absence of Edc3p or Ski2p had no effect on *RPL22B* pre-mRNA levels, suggesting that this transcript is not a major target of Edc3-mediated decapping or of the cytoplasmic exosome. Overexpression of *RPL22A* within this context repressed spliced *RPL22B* levels in all strains but did not affect the sensitivity of the pre-mRNA to any of the decay factors (Fig 1E). Therefore, splicing inhibition by Rpl22p overexpression does not appear to alter the decay fate of cytoplasmic *RPL22B* pre-mRNA.

To ascertain the sensitivity of *RPL22B* pre-mRNA to nuclear decay pathways, we analyzed the impact of the nuclear exosome component deletion strain *rrp6*Δ and of the temperature-sensitive 5’ to 3’ nuclear exonuclease mutant *rat1-1*. No differences in *RPL22B* levels were detected between the wild-type strain and these two mutants at the permissive temperature of 25°C (Fig 1F, lanes 1–3). Shifting wildtype and *rrp6*Δ strains to 37°C for three hours resulted in an observable increase in the levels of pre-mRNA (Fig 1F, lanes 4–5) which was even more pronounced in the *rrp6*Δ strain, consistent with our previous studies [26]. Likewise, shifting *rat1-1* to the non-permissive temperature also resulted in a substantial accumulation of unspliced transcript that also exceeded that seen in wildtype (Fig 1F, lane 6). While these changes at 37°C could be partially attributed to heat shock-induced inhibition of splicing [27,28], the higher levels of unspliced transcript present in the decay mutants in comparison to wildtype suggest that this pre-mRNA is targeted by both 5’ to 3’ and 3’ to 5’ nuclear exonucleolytic degradation mechanisms in heat shock conditions. Interestingly, the levels of spliced *RPL22B* were dramatically reduced in *rrp6*Δ and *rat1-1* at 37°C, whereas the wild-type strain maintained high levels of that transcript (Fig 1F, lanes 4–6). The reason for this effect is unclear, but it is possible that the wild-type strain is able to recover from the initial heat shock and re-establish normal splicing patterns more rapidly than either of the mutant strains. Alternatively, the levels of Rpl22p may be enhanced in nuclear RNA decay factor mutants. Importantly, overexpressing *RPL22A* reduced the levels of spliced transcripts across all strains at both temperatures (Fig 1F, lanes 7–12), but did not appear to influence the susceptibility of the pre-mRNA to degradation by either exonuclease in particular.

In higher eukaryotes the NMD helicase Upf1p is involved in multiple nuclear processes including DNA replication and repair and cell cycle control in addition to several NMD-independent RNA decay pathways (see [29,30] for review). Given our finding that the *RPL22B* pre-mRNA is subject to nuclear RNA degradation, we sought to determine whether the accumulation of the unspliced transcript previously detected in the *upf1*Δ strain is indeed a direct consequence of the lack of NMD activity and not the result of NMD-independent roles of Upf1p. We analyzed three NMD-compromised strains, each lacking one of the three up-frameshift proteins, *upf1*Δ, *upf2*Δ, and *upf3*Δ, along with *xrn1*Δ. All three NMD mutants exhibited a very similar accumulation of the pre-mRNA (Fig 1G), confirming that degradation of the unspliced *RPL22B* pre-mRNA occurs through NMD. Notably, the genetic inactivation of Xrn1p shows a stronger accumulation of pre-mRNA relative to the upstream NMD factors in addition to the accumulation of spliced transcript. When the degradation rate of an RNA transcript is altered, an Xrn1p-dependent RNA buffering mechanism modulates the rate of the RNA synthesis in a compensatory manner [25,31]. Therefore, lack of RNA buffering in the *xrn1*Δ strain to may contribute to the stronger accumulation of these transcripts in that strain relative to the NMD mutants.

Altogether, our results demonstrate that both nuclear and cytoplasmic RNA decay mechanisms are responsible for the degradation of *RPL22B* pre-mRNA, though it is more heavily targeted by the latter. Furthermore, the degradation pathway taken by the transcript does not appear to be influenced by Rpl22p levels.

### Splicing of *RPL22A* pre-mRNA is mildly affected by Rpl22p

The *RPL22* paralogs share a high percentage of nucleotide identity within their protein coding sequences and a high percentage of amino acid identity (S1A Fig). We therefore wondered whether the protein product of *RPL22B* is capable of inhibiting the splicing of the *RPL22B* pre-mRNA. To test this, we exogenously overexpressed intronless *RPL22B* cDNA from a plasmid in either wild-type or NMD mutant strains and analyzed *RPL22B* mRNA splicing patterns by Northern blot. Strikingly, the overexpression of *RPL22B* strongly inhibited the splicing of *RPL22B* pre-mRNA (S1B Fig), demonstrating that the inhibitory property is shared between both protein paralogs. We also tested whether the autoregulation affects the *RPL22A* pre-mRNA by analyzing the splicing of *RPL22A* pre-mRNA upon chromosomal deletion or exogenous overexpression of *RPL22B* or exogenous overexpression of intronless *RPL22A* (S1C–S1E Fig). These experiments revealed a minor repression of spliced *RPL22A* mRNA and slight increase in pre-mRNA levels upon overexpression of either *RPL22* paralog (S1C and S1D Fig), but no affect upon genetic inactivation of *RPL22B* (S1E Fig). Therefore, the *RPL22A* pre-mRNA appears to be mildly susceptible to the autoregulatory activity, though not nearly to the extent seen in *RPL22B*.

### Regulation of *RPL22B* splicing by Rpl22p is mediated by the *RPL22B* intron

**Fig 2:**
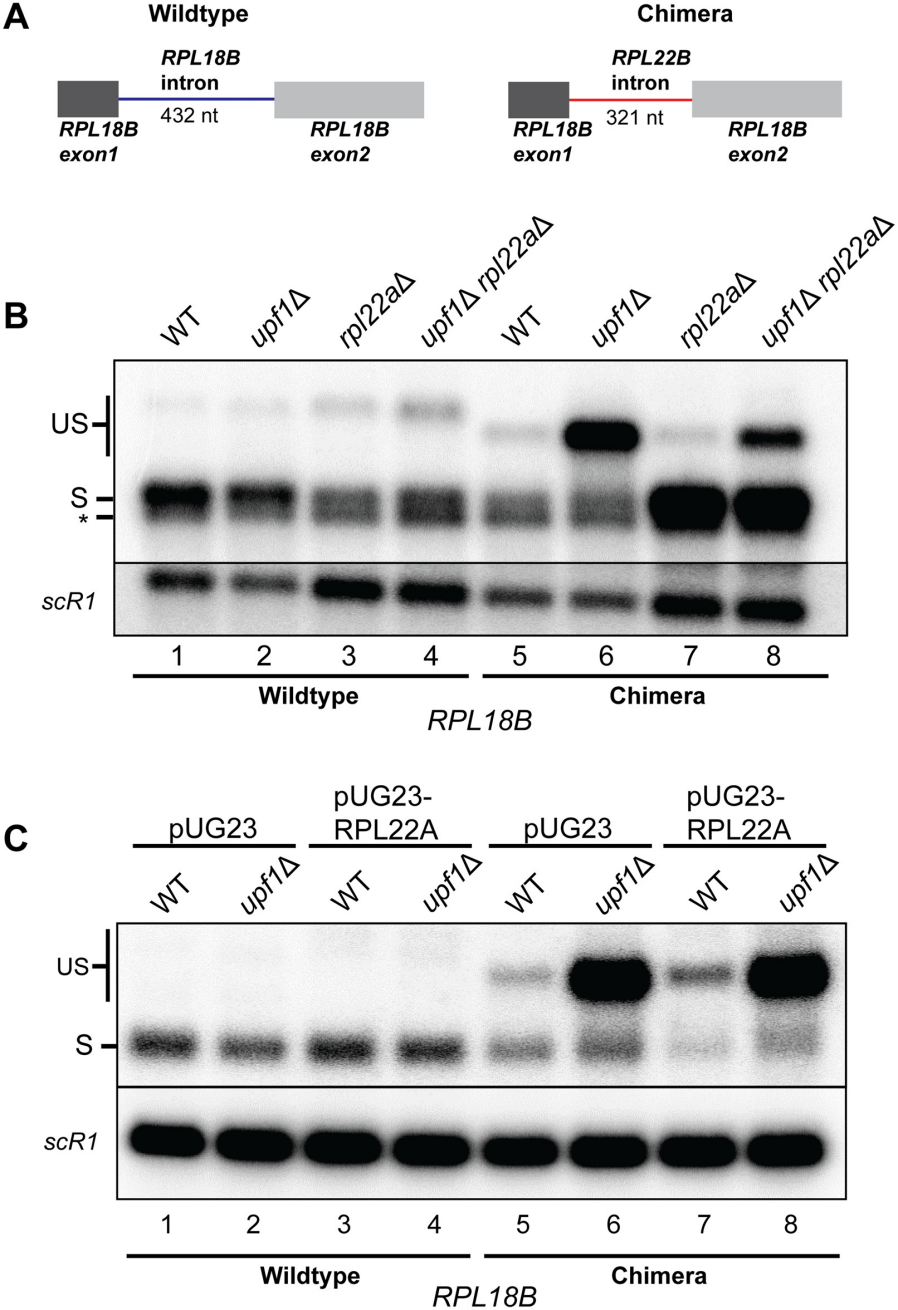
Rpl22p-mediated regulation of splicing is intron-specific. **A.** Schematic of chromosomal wild-type and chimeric *RPL18B* genes. In the chimera, the intron from *RPL18B* was replaced with the intron from *RPL22B* using the *delitto perfetto* approach. **B.** Northern blot detecting the splicing patterns for wild-type and chimeric *RPL22B* transcripts in wild-type and NMD mutant strains with or without the deletion of *RPL22A*. Labeled bands represent the wild-type and chimeric unspliced transcripts (US) and the spliced transcript (S). Transcripts were detected using an *RPL18B* riboprobe that spans the 3’ end of the ORF and the 3’UTR. The asterisk indicates non-specific detection of the *RPL18A* spliced transcript by the *RPL18B* probe. *SCR1* was used as a loading control. **C.** Northern blot detecting the splicing patterns for wild-type and chimeric *RPL22B* transcripts in WT and NMD mutants with or without the overexpression of intronless *RPL22A* from pUG23. Labeling and detection of bands is similar to panel B. *SCR1* was used as a loading control.

Because introns within many RPGs are capable of regulating both inter- and intragenic expression [4], we asked whether the intronic sequence of *RPL22B* is necessary and sufficient for splicing regulation by Rpl22p. To answer this question, we created a chromosomally-expressed chimeric gene at the *RPL18B* locus using the *delitto perfetto* technique [32] in which we replaced the intron of *RPL18B* with the intron from *RPL22B* (Fig 2A). In addition to wild-type strains, these mutations were also made in the context of NMD deficiency, deletion of *RPL22A*, or both (respectively, *upf1*Δ, *rpl22a*Δ, and *upf1*Δ*rpl22a*Δ). As expected, the deletion of *RPL22A* did not increase the splicing efficiency for wild-type *RPL18B* pre-mRNAs, and in fact, appeared to slightly impair splicing (Fig 2B, compare lanes 1 and 2 with 3 and 4). We speculate that this may be an indirect effect of slower growth, as evidenced by the remarkably different splicing patterns for *RPL22B* observed in the slow-growing strains *rpl31a*Δ and *rpl39*Δ compared to wildtype (Fig 1A). More importantly, substitution of the *RPL22B* intron into *RPL18B* reduced the amount of spliced transcript generated from the chimeric pre-mRNA, likely due to splicing inhibition by Rpl22p (Fig 2B, compare lanes 1 and 2 with lanes 5 and 6). In support of this notion, the deletion of *RPL22A* in the context of the chimeric *RPL18B*::*RPL22B* intron gene resulted in a substantial increase in the amount of spliced *RPL18B* transcript (Fig 2B, lanes 7–8), similar to what was observed for the native *RPL22B* transcript (Fig 1C).

To further assess that Rpl22p-mediated regulation can operate *in trans*, we performed the complementary experiment in which *RPL22A* was overexpressed in either wild-type or *upf1*Δ strains expressing native or chimeric *RPL18B*. Rpl22p overexpression had no discernible impact on the splicing of the wild-type *RPL18B* transcript, demonstrating the specificity of this regulatory mechanism (Fig 2C, lanes 1 through 4). By contrast, overexpressing *RPL22A* reduced the levels of spliced chimeric transcript while also increasing the levels of unspliced precursor in the wild-type strain (Fig 2C, compare lanes 5 and 7). Taken together, these experiments suggest that the Rpl22p-mediated splicing regulation of *RPL22B* is mediated by its intron and can operate *in trans* to inhibit the splicing of another transcript harboring the *RPL22B* intron.

### A region within the *RPL22B* intron harboring a stem loop structure is necessary for splicing autoregulation by Rpl22p

**Fig 3:**
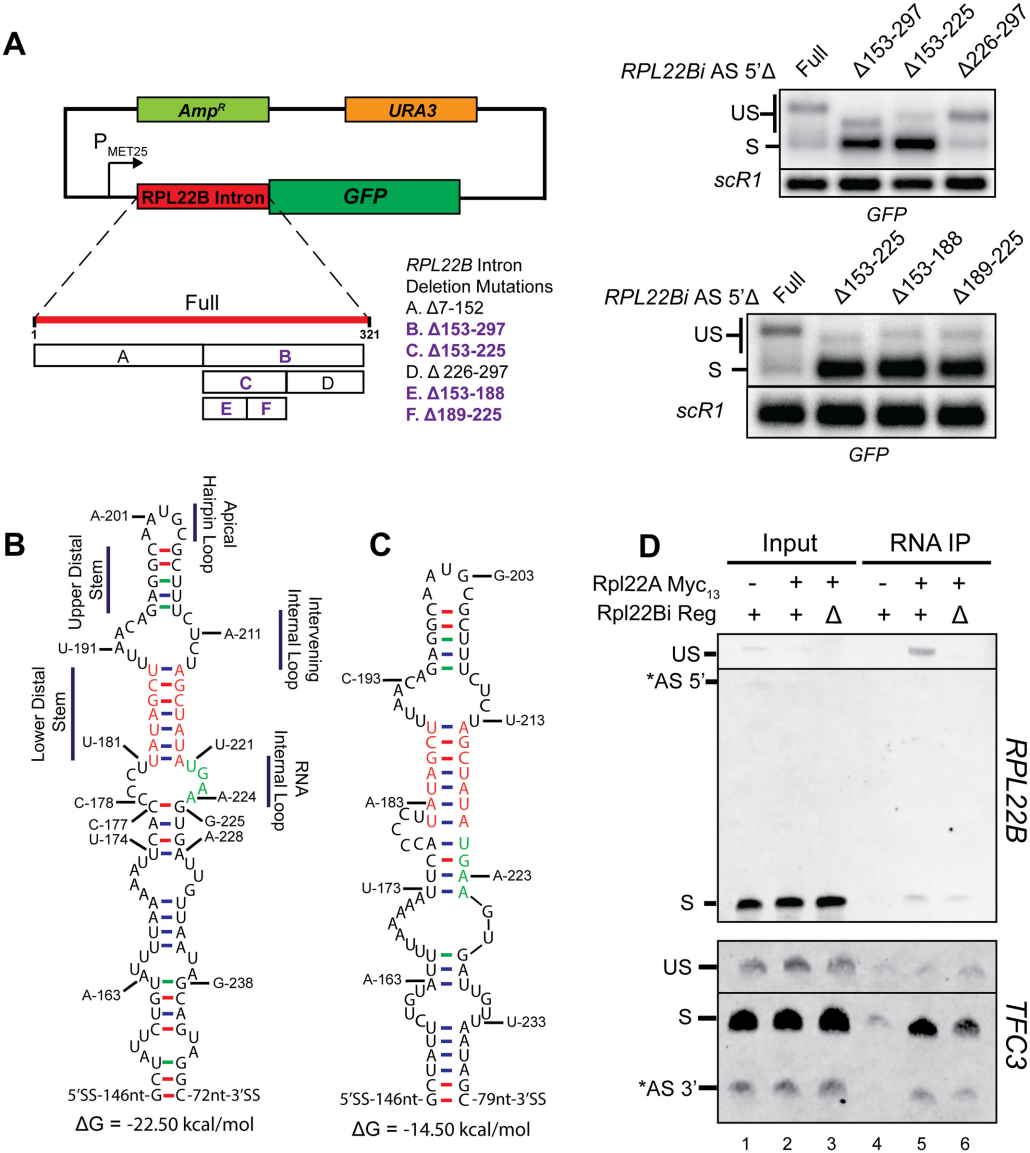
Identification of a regulatory element necessary for binding of Rpl22 to the unspliced *RPL22B* mRNP *in vivo*. **A.** *Left*: Schematic of *RPL22B* intron reporter system based on a pUG35 plasmid backbone. Also indicated are the deletions of the binary search. Deletions highlighted in bold purple font indicate those that result in a loss of splicing inhibition. *Right*: Northern blots demonstrating the loss of splicing inhibition upon deletion of certain segments of the *RPL22B* intron. *SCR1* was used as a loading control. Note that all constructs appearing in these blots do not contain the alternative 5’-splice site. See S1 Text for details. **B.** Structure of the *RPL22B* intron bounded by nucleotides G-153 and C-246 as predicted by Mfold. Regions of interest have been denoted. Nucleotides of the lower distal stem and nucleotides U221 through A224 of the putative RNA Internal Loop were experimentally determined to be important for autoregulatory activity and are colored orange and green, respectively. **C.** Experimentally deduced intronic regulatory element that mediates the inhibition of splicing of the *RPL22B* pre-mRNA. Nucleotide colors from Panel B are retained for comparison. Details describing the experiments leading to the inferred secondary structure are provided in S1 Text. **D.** RNA immunoprecipitation shows the association of Rpl22p with the unspliced *RPL22B* mRNP that requires the regulatory element. *Upper panel*: RT-PCR analysis of *RPL22B* transcripts derived from cells expressing untagged Rpl22Ap (-), Rpl22Ap with the 13-Myc epitope tag (+), and cells with the *RPL22B* intronic regulatory element deleted (Δ) and expressing tagged protein (+). Total input RNA and immunoprecipitated RNA were analyzed from each strain. Labeled bands show the unspliced (US) and spliced (S) species. *Lower panel*: RT-PCR analysis of *TFC3* transcripts performed by similar methods as described for panel A. Bands indicate the unspliced (US) and spliced (S) species. The gel images have been truncated for space considerations. Full, unattenuated versions of the gels can be viewed in S8B Fig.

Previous studies have demonstrated that both direct and indirect autoregulation of genes in yeast are often dependent upon RNA secondary structures [8,11,13]. Based on these precedents, we investigated the presence of a structural regulatory element within the *RPL22B* intron. To simplify the process of manipulating the intron, we first constructed a plasmid-based reporter in which the *RPL22B* intron was attached to a transcript encoding green fluorescent protein (GFP) (Fig 3A). We then utilized site-directed mutagenesis to sequentially bifurcate the intron and assessed loss of splicing inhibition by comparing relative levels of spliced and unspliced reporter transcript by Northern blot (Fig 3A). We excluded the branch point sequence and 5’ and 3’ splice sites from any of the deletions in order to not disrupt the splicing mechanism. Through this approach, we isolated a region spanned by intronic nucleotides 153 through 225 that harbors a prominent secondary RNA structure (as predicted by Mfold [33]) and that was necessary to confer splicing autoregulation. This structure consists of a series of base-paired stems and internal loops closed by an apical hairpin loop (Fig 3B). A more comprehensive description of the binary search approach which led to the identification of this element can be found in S1 Text and S2 Fig.

While this approach allowed us to narrow the window in which the regulatory element resides, it did not provide the necessary resolution to precisely determine the necessary features that enable autoregulation. Therefore, we opted to continue with specific targeted mutations of the putative structure isolated by the binary search. In brief, we found that the identities of the component nucleotides comprising what we refer to as the lower distal stem are inconsequential in governing *RPL22B* regulation, as long as the stem itself is able to form (Fig 3B and 3C [stem nucleotides in red], S4E and S4F Fig). We also determined that efficient autoregulation hinges on the identities of the downstream nucleotides comprising a predicted RNA internal loop, which we later determined experimentally to be paired with upstream nucleotides to form a short stem (highlighted in green in Fig 3B and 3C; see also S5 Fig and S1 Text). The experimentally-deduced structure of the regulatory element is presented in Fig 3C and the details of these experiments are described extensively in S1 Text. We could not demonstrate that this sequence was sufficient to confer splicing inhibition *in vivo* when transposed into a different intron (see S6 Fig, S1 Text and Discussion), possibly because additional intronic elements are required or because the stem-loop is unable to fold correctly in the context of a different transcript. However, a short RNA sequence containing the regulatory element was able to enhance splicing efficiency *in vitro* when added *in trans* to splicing reactions, suggesting that this element was sufficient to titrate Rpl22p in extracts, and thus corresponds to a bona fide binding element for Rpl22p (see below).

### Rpl22p associates with unspliced *RPL22B* mRNPs via the intronic regulatory element

Our previous observations support a splicing-mediated autoregulatory mechanism for *RPL22B*, wherein the changes in splicing efficiency of the *RPL22B* pre-mRNA result from a direct or indirect physical association between the pre-mRNA and protein. To definitively demonstrate this association *in vivo*, we immunoprecipitated the endogenous Rpl22p protein produced from the *RPL22A* locus, using a 13-Myc epitope tag fused to the C-terminus of the Rpl22Ap protein (S8A Fig), followed by RNA analysis (RNA immunoprecipitation or RIP). The RIP was performed on a strain expressing untagged protein as a negative control, the Rpl22Ap-Myc tagged strain expressing wild-type *RPL22B* pre-mRNA, and an Rpl22A-Myc tagged strain expressing the *RPL22B reg*Δ pre-mRNA as an additional negative control. RNA pulldown was followed by RT-PCR analysis.

As expected, analysis of the total input RNA from all three strains indicated that the mature spliced species is the most abundant form of the *RPL22B* transcript in the cell (Fig 3D, upper panel, lanes 1–3). Immunoprecipitation of RNA from the strain expressing untagged protein did not yield any detectable PCR products. However, pulldown of Myc-tagged Rpl22Ap revealed a substantial enrichment of the unspliced *RPL22B* (Fig 3D, lanes 4–5). Because this approach also indirectly precipitates intact ribosomes and any associated translating mRNAs, this enrichment is remarkable in that it clearly exceeds the indirect (ribosome-mediated) association of Rpl22p with the more abundant spliced species that are in the process of being translated. Importantly, the removal of the regulatory element from the *RPL22B* intron eliminated this enrichment for the pre-mRNA in the pulldown (Fig 3D, lane 6), confirming that the regulatory element is required for *in vivo* association between Rpl22p and the unspliced *RPL22B* mRNPs. To further ensure that this interaction is specific for the *RPL22B* pre-mRNA, we also analyzed Rpl22p association with *TFC3* by RT-PCR. As anticipated, pulldown of tagged Rpl22p mostly detected a ribosome-mediated interaction with the canonically spliced *TFC3* transcript, a preference that was unchanged by the removal of the *RPL22B* intronic regulatory element (Fig 3D, bottom panel). We conclude that the *RPL22B* intronic regulatory element is necessary to mediate an interaction between the Rpl22 protein and the unspliced *RPL22B* mRNP *in vivo*.

We next asked whether a structure similar to the identified regulatory element may be governing the interaction between Rpl22p and the large ribosomal RNA (rRNA) in the intact ribosome, as this would represent a logical extension of the protein’s natural interactions with rRNA. Examination of published crystallographic and computational structural data [34,35] describing the large ribosomal subunit (LSU) reveals that Rpl22p is an external ribosomal protein that indeed appears to be in contact with a stem loop of the LSU rRNA (S9A Fig). This structure bears no discernible similarity to the regulatory element identified by our studies (Figs S9B and 3C). It does, however, appear to comply with the generalized human Rpl22 RNA-binding motif that was previously identified by SELEX [36] (see Discussion), suggesting that the mRNA binding propensity of mammalian Rpl22 stems from its native interactions with rRNA. The fact that the *RPL22B* regulatory element identified in *S*.*cerevisiae* does not resemble these sequences or structures may suggest that the RNA binding specificity of the Rpl22p protein has uniquely evolved in the yeast system or that the binding of Rpl22p to the unspliced *RPL22B* mRNP may be indirect and involve a mediating factor. Alternatively, we cannot rule out that the yeast intronic regulatory element might adopt a fold similar to that found in the large rRNA but that this cannot be predicted from its secondary structure.

### Regulation of *RPL22B* does not depend on cytoplasmic localization

**Fig 4:**
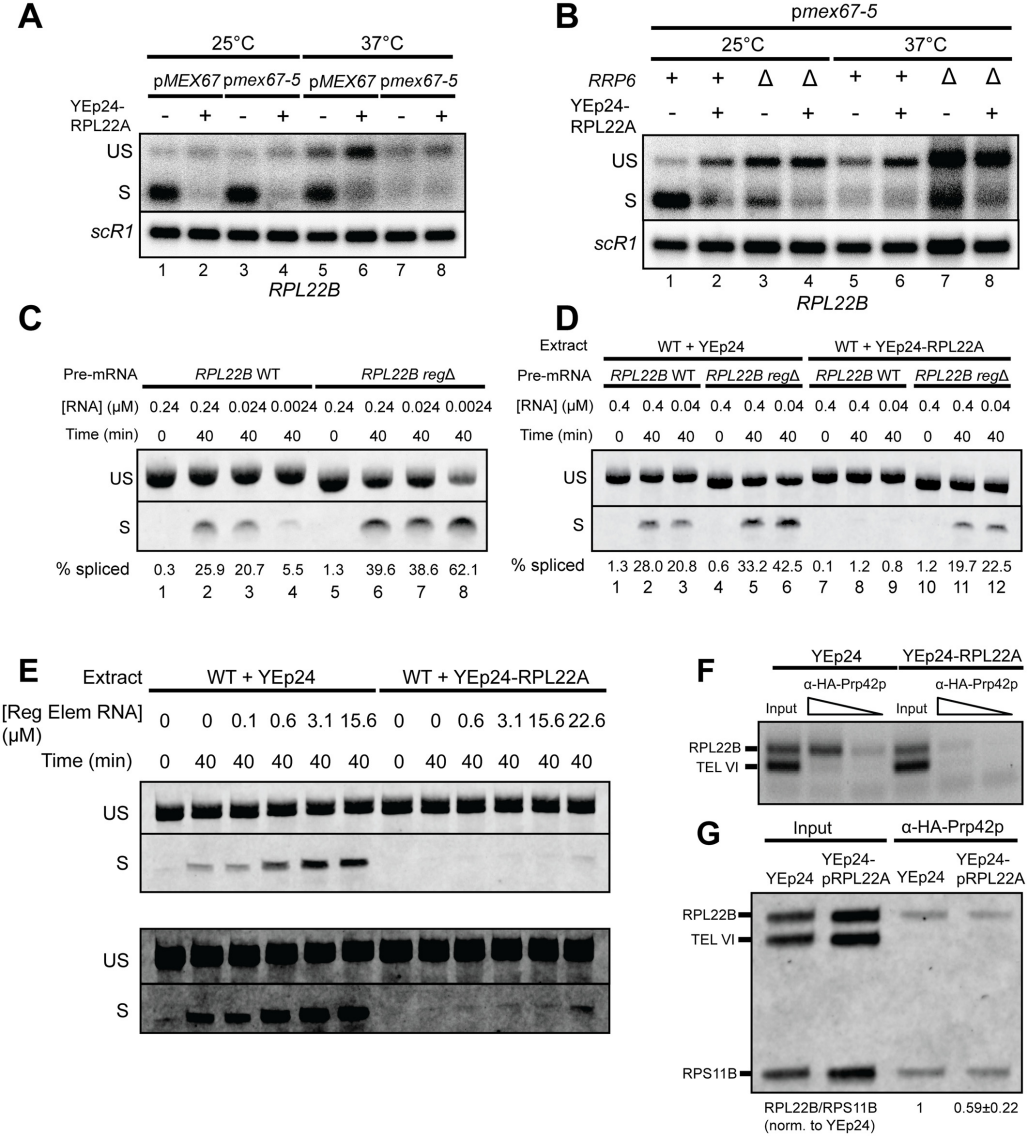
Inhibition of *RPL22B* splicing by Rpl22p in the nucleus can be reproduced *in vitro* and may involve a repression of the co-transcriptional U1 snRNP recruitment *in* vivo. **A.** Northern blot analysis of *RPL22B* mRNA expression in *mex67*Δ strains carrying plasmids expressing either wild-type *MEX67* or the temperature sensitive *mex67-5* allele and either the empty Yep24 vector or the *RPL22A* overexpression plasmid at permissive (25°C) and non-permissive (37°C) temperatures. The unspliced (US) and spliced (S) RNA species are shown. *SCR1* was used as a loading control. **B.** Northern blot analysis of the *mex67-5* strain similar to panel A with the addition of the genetic inactivation of *RRP6*. Detection methods and transcript labels are similar to panel A. **C.** RT-PCR analysis of *in vitro* splicing reactions in wild-type splicing extract. Decreasing amounts of *RPL22B* wild-type and *reg*Δ pre-mRNA were added to splicing reactions and incubated at 25°C for 40 minutes. The RNA was then purified and analyzed by RT-PCR. Labeled bands indicate the unspliced (US) and spliced (S) species. The gel image has been truncated for space considerations; the uncut image can be viewed in S13 Fig. **D.** RT-PCR analysis of splicing reactions utilizing wild-type and *RPL22A* overexpression splicing extracts. *RPL22B* wild-type and *reg*Δ pre-mRNA were added to splicing reactions at the indicated concentrations and incubated at 25°C for 40 minutes. The RNA was then purified and analyzed by RT-PCR. Labeled bands indicate the unspliced (US) and spliced (S) species. The gel image has been truncated for space considerations; the uncut image can be viewed in S14 Fig. Note also the slightly smaller size of the *reg*Δ pre-mRNA versus wild-type due to the deletion of 14 nucleotides. **E.** RT-PCR analysis of *RPL22B in vitro* splicing reactions in the presence of short RNA transcripts containing the regulatory element with light (upper panels) and dark (lower panels) exposures. Splicing extracts were prepared from wild-type yeast carrying empty YEp24 or an *RPL22A* overexpression vector. All splicing reactions contained 0.005 μM *RPL22B* pre-mRNA. Increasing amounts of regulatory element RNA were added to splicing reactions to the final concentrations shown. Splicing reactions were incubated at 25°C for 40 minutes, after which the RNA was purified and used for RT-PCR. Labeled bands indicate the unspliced (US) and spliced (S) species. See S1 Table for primers used to generate the regulatory element RNA transcript. The gel images have been truncated for space considerations; the uncut images can be viewed in S7 Fig. **F.** Analysis of U1 snRNP recruitment at the *RPL22B* locus by chromatin immunoprecipitation. Ethidium bromide stained gel showing PCR products amplified from chromatin obtained from Prp42-HA ChIP or the corresponding input chromatin. The ChIP chromatin concentration was titrated for analysis. DNA was amplified using a multiplexed PCR reaction containing primer sets for the intronic regions of *RPL22B* (target gene) and telomeric region of chromosome VI (TEL VI, ChIP negative control). **G.** Same as E, but Cy3-labeled PCR products were amplified from chromatin obtained from Prp42-HA ChIP or the corresponding input chromatin. DNA was amplified using a multiplexed PCR reaction containing primer sets for the intronic regions of *RPL22B* (target gene) and *RPS11B* (ChIP positive control) and the telomeric region of chromosome VI (ChIP negative control). The quantification of repression of Prp42p association with *RPL22B* chromatin upon overexpression of *RPL22A* in the ChIP samples is displayed below the gel image. Within each ChIP sample the *RPL22B* PCR signal was normalized internally to the *RPS11B* PCR signal. These ratios were then normalized to the ratio for YEp24, providing a measurement of the decrease of Prp42p association with *RPL22B* upon *RPL22A* overexpression. The value shown indicates mean ± 1 standard deviation for two biologically independent ChIP experiments.

The apparent “inhibition” of splicing that we detected upon Rpl22 overexpression would also be predicted if Rpl22p expedites the nuclear export of the pre-mRNA and sequesters it out of reach of the spliceosome, a scenario made possible by the trafficking of ribosomal proteins in and out of the nucleus during ribosome maturation [37,38]. To test whether Rpl22p-mediated autoregulation operates when the *RPL22B* unspliced pre-mRNA is constrained in the nucleus, we analyzed the splicing of *RPL22B* pre-mRNA in strains containing a temperature-sensitive mutation of the mRNA export factor *MEX67* [26]. As expected, overexpression of *RPL22A* resulted in the repression of spliced *RPL22B* mRNA in strains expressing either wild-type *MEX67* or the mutant allele *mex67-5* at the permissive temperature (Fig 4A, lanes 1–4). However, the levels of spliced transcript were repressed in the *mex67-5* mutant upon shifting to the non-permissive temperature with or without *RPL22A* overexpression (Fig 4A lanes 7–8 and Fig 4B lanes 5–6), suggesting that this transcript might be subject to turnover by the nuclear exosome in these conditions. Indeed, genetic inactivation of Rrp6p resulted in increased levels of both spliced and unspliced *RPL22B* mRNA (Fig 4B, lane 7), confirming that these transcripts are degraded by the nuclear exosome when nuclear export is defective, consistent with our previous findings [26]. Importantly, overexpression of *RPL22A* in these conditions resulted in a repression of the spliced *RPL22B* transcript (Fig 4B, lane 8). This suggests that the pre-mRNA is subject to autoregulation even when it is localized to the nucleus and that the regulatory mechanism functions via inhibition of splicing.

To further confirm that the autoregulatory mechanism functions at the level of splicing regulation, we investigated whether inhibition of *RPL22B* pre-mRNA splicing could be recapitulated *in vitro*, where the transcript is continuously exposed to the splicing machinery. A wild-type version of the *RPL22B* unspliced transcript and a mutant lacking intronic nucleotides 178 through 191 (*reg*Δ), which removes a section of the stem necessary for autoregulation and mRNP binding *in vivo* (see above) were generated by *in vitro* transcription. We assembled *in vitro* splicing reactions by adding these pre-mRNA substrates at various concentrations to splicing extracts derived from wild-type cells, and we determined the extent of splicing of the exogenous *RPL22B* by RT-PCR (Fig 4C and 4D). Strikingly, we found that the splicing efficiency of the wild-type pre-mRNA substrate in wild-type extracts was dependent on the concentration of the pre-mRNA, with the proportion of spliced mRNA decreasing progressively as the concentration of the splicing substrate was reduced (Fig 4C, lanes 1–4). We hypothesized that this might be due to the saturation of the relatively small amount of free Rpl22p present in the extracts by the pre-mRNA at higher substrate concentrations, leaving the majority of the pre-mRNA available to be acted upon by the spliceosome; at lower pre-mRNA substrate concentrations there is sufficient free Rpl22p in the splicing reaction to efficiently inhibit splicing. In agreement with this hypothesis, the splicing of the *reg*Δ substrate remained efficient with decreasing substrate concentration (Fig 4C, lanes 5–8), also demonstrating that splicing inhibition does not occur in the absence of the regulatory element *in vitro*. To confirm that excess Rpl22p can inhibit the splicing of *RPL22B in vitro*, we analyzed the splicing of both wild-type and *reg*Δ substrates in splicing extracts derived from cells overexpressing Rpl22p. As predicted, the overexpression of Rpl22p resulted in a nearly complete suppression of splicing of the wild-type pre-mRNA substrate *in vitro*. (Fig 4D, lanes 7–9). Importantly, deletion of the regulatory element restored splicing to a level comparable to that of the wild-type substrate in wild-type splicing extracts (Fig 4D, compare lanes 1–3 with lanes 10–12). Thus, the intronic regulatory element enables Rpl22p to effectively inhibit the splicing of the *RPL22B* pre-mRNA *in vitro*, and the overall efficiency of splicing is influenced by the relative levels of pre-mRNA and Rpl22p protein. Moreover, splicing of the pre-mRNA is inhibited *in vitro* when the spliceosome cannot be spatially separated from the substrate by cellular relocalization, arguing against a model involving the expedited export of the pre-mRNA to the cytoplasm *in vivo*.

### The addition of a short RNA fragment harboring the regulatory element sequences enhances *RPL22B* pre-mRNA splicing *in vitro*

The transposition of the *RPL22B* intronic regulatory element into the *RPS21A* intron did not confer splicing-based autoregulation of the *RPS21A* pre-mRNA (S6 Fig), possibly because this RNA structure does not fold properly in the context of the foreign intron and therefore does not bind Rpl22p. However, we hypothesized that a shorter RNA transcript containing only the regulatory element sequence might effectively sequester Rpl22p, resulting in increased splicing efficiency *in vitro*. To investigate this further, we repeated the *in vitro* splicing reactions utilizing low concentrations of *RPL22B* pre-mRNA, conditions which were shown above to result in low splicing efficiency (Fig 4C). We then added increasing concentrations of a 147 nucleotides-long RNA transcript harboring the regulatory element to test whether the addition of this transcript would enhance pre-mRNA splicing. This experiment showed that the regulatory element RNA sequence alone is sufficient to increase splicing of the pre-mRNA in a concentration-dependent manner (Figs 4E and S7). Importantly, splicing reactions in extracts derived from yeast overexpressing Rpl22p required comparably higher concentrations of the regulatory element RNA in order for splicing efficiency to be improved (Figs 4E and S7), further showing that splicing efficiency is directly influenced by the amount of Rpl22p protein unbound to the regulatory element. Taken together, these results strongly suggest that, at least *in vitro*, the isolated regulatory element is sufficient to directly or indirectly bind and sequester Rpl22p so that the protein is unable to inhibit splicing of the *RPL22B* pre-mRNA.

### Rpl22p overexpression reduces co-transcriptional recruitment of U1 snRNP to the *RPL22B* locus

Previous chromatin immunoprecipitation (ChIP) studies have shown that *RPL22B* is one of many intron-containing chromosomal loci to which the U1 snRNP is recruited co-transcriptionally [39]. In light of these findings, we hypothesized that the repression of *RPL22B* pre-mRNA splicing may be due in part to the interference of co-transcriptional U1 recruitment. To test this, we performed ChIP on yeast strains expressing HA-tagged versions of the U1 snRNP protein Prp42p to determine whether the overexpression of Rpl22p impedes the association of the protein with chromatin at the *RPL22B* locus. Indeed, we found that yeast cells carrying the *RPL22A* overexpression vector exhibited a slightly repressed enrichment of Prp42p at the *RPL22B* locus relative to cells transformed with the control vector (Fig 4F). As a normalization control, we also examined the recruitment of Prp42p to *RPS11B*, another RPG which demonstrates strong co-transcriptional U1 recruitment [39] and should be relatively unaffected by Rpl22p overexpression (Fig 4G). Quantitative analysis showed that Rpl22p overexpression reduced U1 snRNP co-transcriptional recruitment by about 40%. These experiments suggest that Rpl22p inhibits U1 snRNP recruitment to the *RPL22B* locus. We carefully note, however, that this result only gives a partial explanation for the mechanism of splicing inhibition, as the repressive effect of Rpl22p overexpression on *RPL22B* pre-mRNA splicing *in vivo* and *in vitro* (Figs 1D–1G, 4D and 4E) appears more substantial than the minor repression of U1 recruitment detected here.

### Rpl22 paralog identity does not dictate cellular sensitivity to ribosome inhibitors

In wild-type yeast cells the proportion of ribosomes harboring the Rpl22A paralog exceeds the proportion harboring the Rpl22B paralog by a factor of more than 15 [18], suggesting that the *A* paralog plays the frontline role for actively-translating ribosomes. The dearth of Rpl22Bp in wild-type ribosomes begs the question of whether yeast have evolved to specifically regulate the *RPL22* paralog composition of ribosomes, perhaps to influence cell fitness when challenged with antagonistic conditions such as chemical stressors. We therefore performed an antibiotic screen utilizing an array of chemicals known to target the ribosome, including anisomycin, paromomycin, puromycin, neomycin, and verrucarin A [40,41]. We assessed the growth of wild-type and *rpl22a*Δ strains transformed with an empty vector or with plasmids overexpressing either intronless *RPL22A* or *RPL22B*. In general, growth of wild-type cells was unimpeded by the antibiotic concentrations tested and was not affected by the overexpression of either paralog (S10 Fig). The *rpl22a*Δ strain demonstrated the slowest growth in nearly every condition. However this growth defect could be rescued by the overexpression of either *RPL22* paralog, except in the case of verrucarin A (S10 Fig). Interestingly, *rpl22a*Δ demonstrated more robust growth in the presence of this chemical compared to the wildtype, possibly due to a defect in ribosomal subunit assembly that may confer hyperresistance to this antibiotic [40]. Overall, our observations do not support the notion of Rpl22p paralog composition influencing cellular sensitivity to the particular ribosome inhibitors and concentrations tested, though we note that our screen was limited in scope and certainly does not preclude this possibility.

### Inhibition of *RPL22B* pre-mRNA splicing promotes down-regulation of mature mRNA during MMS-Induced DNA damage

**Fig 5:**
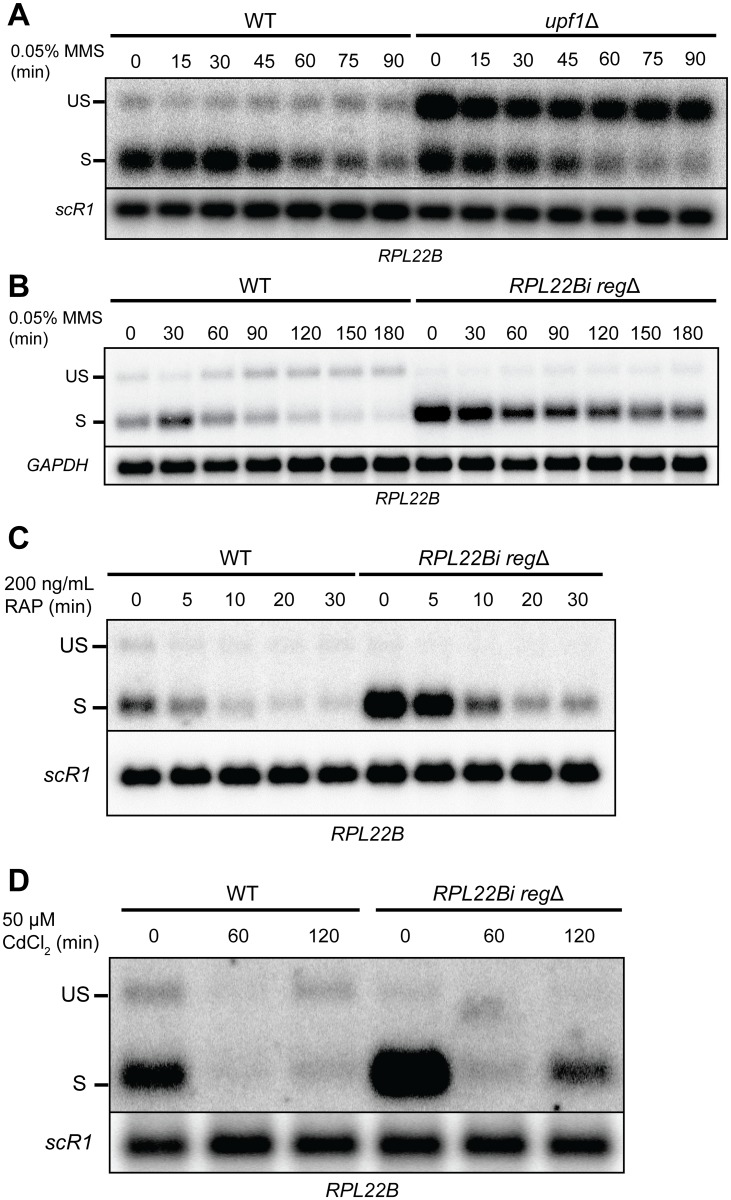
Inhibition of Rpl22p-mediated splicing autoregulation attenuates downregulation of *RPL22B* in response to MMS-induced DNA damage but not rapamycin treatment. **A.** Northern blot analysis of splicing kinetics for *RPL22B* in wild-type and *upf1*Δ strains in response to MMS-induced DNA damage. Cells were grown to exponential phase in YPD media and then shifted to YPD media containing 0.05% MMS for 90 minutes. *RPL22B* transcripts were detected with an *RPL22B* 5’UTR riboprobe. Shown are the unspliced (US) and spliced (S) species. *SCR1* was used as a loading control. **B.** Northern blot analysis of splicing kinetics for *RPL22B* in wild-type yeast and in a strain in which the regulatory element has been removed from the *RPL22B* intron (*RPL22Bi reg*Δ). Cells were grown in YPD containing 0.05% MMS for 180 minutes. Bands are labeled similarly to panel A. *GAPDH* was used as a loading control. **C.** Northern blot analysis of splicing kinetics for *RPL22B* in wild-type yeast and in a strain in which the regulatory element has been removed from the *RPL22B* intron (*RPL22Bi reg*Δ). Cells were grown in YPD containing 200 ng/mL rapamycin for 30 minutes. Detection methods and labels are similar to panels A-B. **D.** Northern blot analysis of *RPL22B* splicing in WT and *RPL22Bi reg*Δ strains when were grown in YPD containing 50 μM CdCl_2_ for 120 minutes. Detection methods and labels are similar to panels A-C.

Splicing-specific microarray studies have revealed that ribosomal protein genes undergo changes in pre-mRNA processing in response to a variety of environmental stresses, including amino acid starvation, growth in non-fermentable carbon sources, osmotic stress and heat shock [27,42]. Earlier experiments have also shown that the transcript levels of many RPGs are repressed by exposure to methane methyl-sulfonate (MMS) [1], an alkylating agent that is thought to stall DNA replication fork movement. In agreement with these results, we observed a tapering of spliced *RPL22B* mRNA levels in cells treated with 0.05% MMS (Fig 5A). Surprisingly, MMS-treatment of the NMD-deficient strain *upf1*Δ resulted in a substantial accumulation of *RPL22B* pre-mRNA that persisted even as the mature transcript gradually decreased (Fig 5A). Based on this result, we hypothesized that the repression of mature *RPL22B* transcript during MMS-induced DNA damage relies primarily on the inhibition of splicing rather than transcriptional repression, which may in turn depend on the regulatory mechanism that we identified herein.

To test whether the *RPL22B* intronic regulatory element regulates the splicing of *RPL22B* pre-mRNA during DNA damage, we treated a strain harboring a chromosomal deletion of the intronic regulatory element (*RPL22Bi reg*Δ) with 0.05% MMS for three hours and compared its splicing patterns to those of the wild-type strain. As described above, the wild-type strain exhibited a persistent accumulation of unspliced transcript as the mature transcript decreased over the course of the treatment. By the end of the treatment the proportion of unspliced transcript was noticeably higher than the proportion of spliced transcript (Fig 5B). However, removal of the regulatory element largely negated this effect—the pre-mRNA did not accumulate over the three-hour treatment and the spliced transcript remained the predominant species throughout the time course of the experiment (Fig 5B). This difference was also apparent when comparing quantitatively the reduction of the amount of spliced transcript triggered by the MMS treatment within each strain. Whereas a 3 hour treatment reduced spliced *RPL22B* to c.a. 18% of its pre-treatment levels in the wild-type strain, deletion of the regulatory element resulted in an attenuated reduction to only 58% of its pre-treatment levels (S11A Fig). Thus, the inhibition of splicing through the *RPL22B* intronic regulatory element appears to play a major role in maximizing the cell’s ability to suppress levels of mature *RPL22B* transcript during DNA damage.

To determine whether the Rpl22 protein is necessary for regulation in DNA damage conditions, we repeated the MMS treatment experiment in the *rpl22a*Δ strain. In the wild-type strain, we observed a shift in splice patterns in which the levels of unspliced transcript becomes comparable to the spliced species after three hours of MMS treatment (S11B Fig). By contrast, removal of the intronic regulatory element (*cis*, *RPL22Bi reg*Δ) or deletion of *RPL22A* (*trans*, *rpl22a*Δ) prevented this shift and resulted in the spliced species remaining the predominant form (S11B Fig). These results demonstrate that the down-regulation of mature *RPL22B* transcript levels depends at least in part on the ability to inhibit splicing of the pre-mRNA through the intronic regulatory element.

To test whether the down-regulation of either of the mature *RPL22* transcripts impacts cell growth in these conditions, wild-type yeast carrying an empty vector or overexpression plasmids for either *RPL22A* or *RPL22B* were monitored for cell growth in a range of MMS concentrations. Importantly, because the exogenous *RPL22* transcripts are intronless, they are unaffected by the inhibitory mechanism. Cell growth was similarly impeded by MMS treatment in a concentration-dependent manner for all three strains (S11C Fig), suggesting that the inability to repress of this specific protein *per se* is not detrimental to cell fitness when grown in the presence of MMS. However, we propose that the biological significance of *RPL22B* pre-mRNA splicing inhibition in MMS-induced DNA damage may be rooted in the liberation of the spliceosomal machinery rather than the repression of the *RPL22B* transcript itself (see Discussion).

Rapid changes in yeast pre-mRNA levels are known to occur not only in MMS-induced DNA damage but also in a variety of environmental stress conditions. For example, amino acid depletion results in the inhibition of splicing and a subsequent accumulation of RPG pre-mRNAs [42]. Additionally, inhibition of the target of rapamycin complex 1 (TORC1) via rapamycin treatment has been shown to suppress RPG expression by preventing the phosphorylation of effector proteins that modulate RPG transcription [43]. *RPL22B* was found to be one of many RPGs whose transcript levels are decreased upon rapamycin treatment [27]. For these reasons we investigated whether the suppression of this transcript during TORC1 inhibition uses a mechanism akin to the autoregulation of splicing described during MMS-induced DNA damage. To address this question, we analyzed the relative levels of spliced and unspliced *RPL22B* transcript in WT and *RPL22Bi reg*Δ cells after rapamycin treatment. As expected, the levels of spliced transcript were reduced in both strains following rapamycin treatment (Fig 5C). However, in contrast to what was observed during MMS treatment, the unspliced transcript did not accumulate during rapamycin treatment and the spliced transcript remained the dominant species (Fig 5C). Thus, the regulation of *RPL22B* in the presence of rapamycin appears to rely primarily on transcriptional repression, whereas down-regulation during MMS-induced DNA damage primarily involves auto-inhibition of pre-mRNA splicing.

### Disruption of rRNA transcription causes inhibition of *RPL22B* pre-mRNA splicing

The observation that *RPL22B* pre-mRNA splicing appeared to be inhibited upon genetic inactivation of *RPL31A* and *RPL39* (Fig 1A) suggests that this effect might be due to defective ribosome assembly in these mutants. A reduction in the efficiency of ribosome assembly would increase the population of free ribosomal proteins or pre-ribosomal protein complexes, resulting in excess amounts of free or complexed Rpl22p available to inhibit the splicing of the pre-mRNA. Similar results might be expected in other conditions that impede the assembly of ribosomes, including the down-regulation of rRNA transcription. To test this hypothesis, we took advantage of the ability of cadmium to suppress rRNA transcription by RNA polymerase I [44]. Treatment of wildtype and *RPL22Bi reg*Δ strains with 50 μM CdCl_2_ for two hours resulted in a reduction in the levels of 35S rRNA as well as other intermediates in the rRNA biogenesis pathway (S12 Fig). Following an initial decrease of all *RPL22B* transcripts at 60 minutes, analysis of *RPL22B* transcripts at 120 minutes revealed a clear inhibition of pre-mRNA splicing (Fig 5D, lanes 1 through 3). This inhibition was not observed in the absence of the regulatory element, resulting in the spliced species remaining the predominant form after 120 minutes (Fig 5D, lanes 4 through 6), similar to the behavior observed during MMS treatment. Thus, the disruption of rRNA transcription can inhibit *RPL22B* pre-mRNA splicing, presumably by suppressing *de novo* ribosome biogenesis and increasing cellular concentrations of free or complexed Rpl22p. Overall, these results show that autoregulation of *RPL22B* splicing is a major mechanism that contributes to the downregulation of *RPL22B* in conditions which are known to decrease ribosome biogenesis.

## Discussion

**Fig 6:**
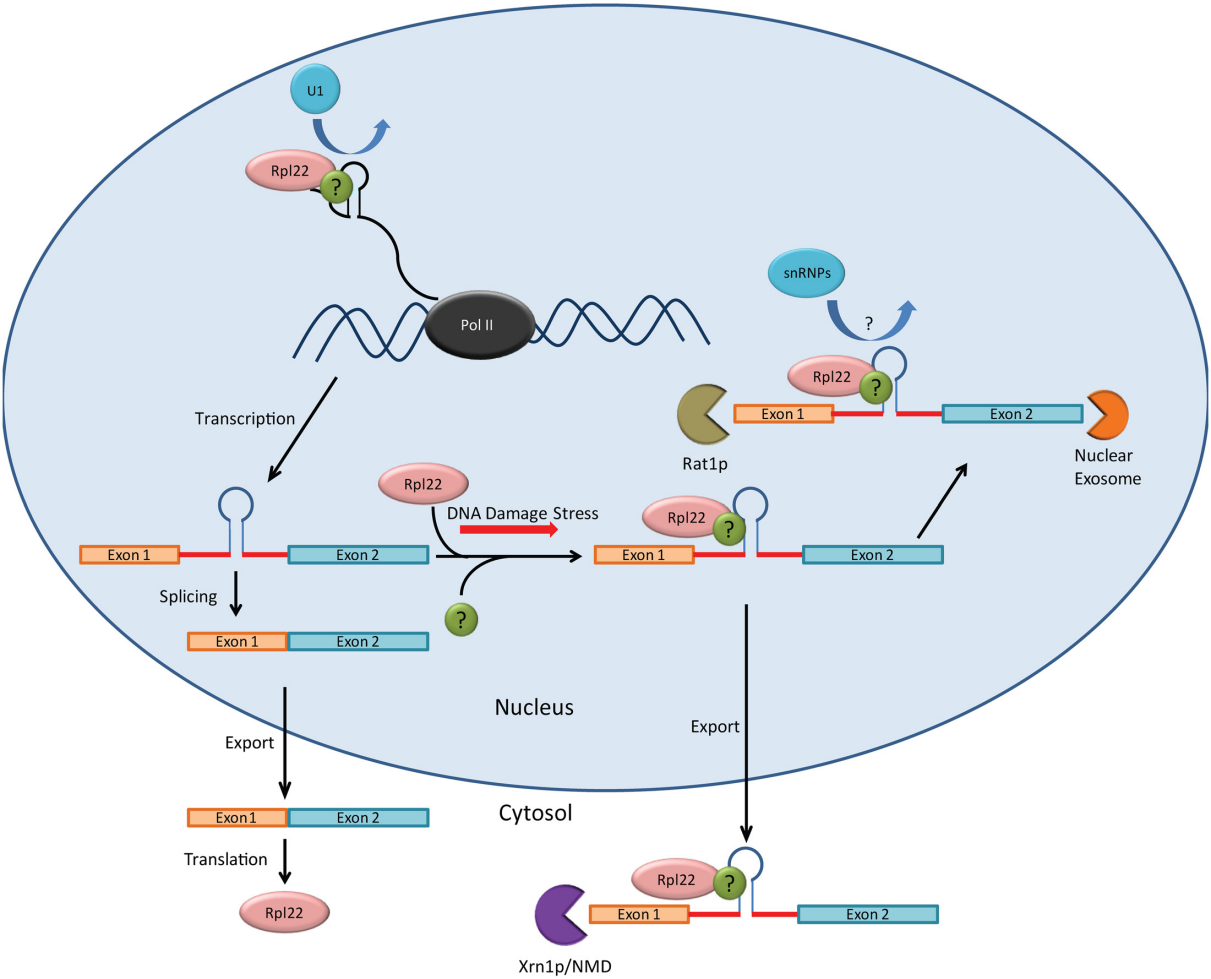
Model for the autoregulation of *RPL22B*. *RPL22B* pre-mRNA contains an intronic regulatory element, depicted here by a simplified stem loop, to which the Rpl22 protein is able to directly or indirectly bind to associate with the unspliced mRNP. This association inhibits splicing of the pre-mRNA, potentially by prohibiting binding of one or more spliceosomal snRNPs, which is then either degraded by nuclear exonucleases or exported to the cytoplasm and degraded by NMD. Splicing inhibition is exacerbated during DNA damage stress. The green protein labeled with the black question mark represents potential binding factors that may facilitate an indirect interaction between Rpl22p and the *RPL22B* unspliced mRNP.

In this study, we have shown that *S*.*cerevisiae* utilizes an autoregulatory system to control the cellular levels of the *RPL22B* mRNA. The Rpl22 protein directly or indirectly recognizes and binds to an RNA structure present in the *RPL22B* intron, resulting in inhibited splicing of the pre-mRNA. Deletion of the regulatory element results in a substantial increase in the levels of spliced *RPL22B* mRNA at steady-state, indicating that *RPL22B* is subject to continuous repression by its own protein product. Altogether, our results illustrate a model in which the binding of Rpl22p to the *RPL22B* pre-mRNA interferes with splicing of the transcript. The pre-mRNA is then susceptible to degradation by exonucleases in the nucleus or to export and 5’-to-3’ degradation by Xrn1p, likely as the final step of the NMD pathway (Fig 6). This regulatory activity constitutes an extra-ribosomal function for Rpl22p that serves to regulate the cellular levels of the protein and, by extension, the Rpl22p paralog composition of ribosomes.

Because we analyzed Rpl22p binding to the *RPL22B* pre-mRNA i*n vivo*, our experiments did not determine whether Rpl22p binds the pre-mRNA as a free protein or as part of a larger RNP complex, including possibly the entire pre-60S LSU, the latter being possible due to Rpl22p’s external position on the ribosome [45]. However, because *RPL22* overexpression led to a decrease in pre-mRNA splicing presumably without concomitant stoichiometric increases in the expression of other RPGs, we speculate that the binding to the pre-mRNA could involve either free protein or in association with other proteins, but likely not the entire LSU. Our results also revealed inhibition of pre-mRNA splicing following the disruption of rRNA transcription (Fig 5D), presumably by impeding ribosome assembly and relinquishing free or complexed Rpl22p. In addition to Rpl22p, this model would also predict that the extra-ribosomal functions of other ribosomal proteins might become more prevalent in these conditions.

Finally, our results suggest that the co-transcriptional recruitment of the U1 snRNP to the *RPL22B* locus is at least partially inhibited by the increased presence of Rpl22p, which may explain some of the loss of splicing of the *RPL22B* pre-mRNA (Fig 4F and 4G). However, we are careful to note that this mechanism may not fully account for the entirety of the splicing inhibition. In addition, *in vitro* splicing experiments suggest that splicing can also be inhibited post-transcriptionally (Fig 4C and 4D), in agreement with evidence suggesting that the splicing of *S*. *cerevisiae* pre-mRNAs containing short second exons (<500 nt; the second exon of *RPL22B* is 377 nt in length) is primarily post-transcriptional [46]. We speculate that post-transcriptional splicing inhibition may entail the prevention of one or more of the spliceosomal snRNPs from effectively binding the pre-mRNA (Fig 6).

The intronic RPG regulation study conducted by Parenteau and colleagues found that the choromosomal deletion of the *RPL22A* intron resulted in a >10-fold repression of *RPL22B* mRNA levels and a 75% increase in *RPL22A* mRNA levels [4]. In agreement with these findings, our overexpession experiments with intronless *RPL22A* likewise resulted in a substantial repression of mature *RPL22B* (Fig 1). Furthermore, the chromosomal deletion of the *RPL22B* intron triggered a >6-fold increase in mature *RPL22B* transcript levels [4]. Based on our results, we can confidently attribute this observation to the role of the *RPL22B* intron in regulating the expression of the mature transcript through the splicing-mediated inhibitory mechanism that we have identified herein. Thus, our results are very consistent with previous observations and provide additional mechanistic insight into the regulatory activity of *RPL22*.

### A unique regulatory element for a familiar purpose

The structure and composition of the *RPL22B* regulatory element and the associated regulatory mechanism that we have identified in this study is reminiscent of other cases of ribosomal protein autoregulation, some of which were shown to be based on splicing. A classical example of a yeast RPG autoregulatory system was identified for *RPL30* by Eng and Warner [8]. In this case, the regulatory element pairs the nucleotides of the intronic 5’ splice site with nucleotides further upstream on the pre-mRNA transcript, thereby sequestering the 5’ splice site into a secondary structure. The Rpl30 protein stabilizes this configuration by binding to an internal loop structure immediately adjacent to the 5’-splice site, thereby preventing the U2 snRNP from accessing the substrate [47,48]. A similar mechanism has been elucidated for *RPS14B*, in which the Rps14 protein was found to bind the *RPS14B* pre-mRNA in which the 5’ splice site was likewise predicted to fold into a secondary structure, repressing its expression [9], although the question of whether this directly impacts pre-mRNA splicing was not addressed. Other studies focusing on the mechanisms of 3’SS selection have found that that the availability of this splice site, and therefore splicing propensity, is largely dictated by its sequestration into pre-mRNA secondary structures, which in turn is influenced by temperature [49]. By contrast, the regulatory element identified herein for *RPL22B* does not include any of the three primary sites required for spliceosome assembly, arguing against a direct “shielding” of splice sites from access by the spliceosome. However, we cannot rule out the possibility that the formation of this element impacts the folding of the remainder of the intron, which may in turn dictate the inclusion of these sites within more complex structures. Alternatively, it is possible that the binding of the protein induces a conformational change in the *RPL22B* mRNP that disfavors splicing by steric hindrance. Thus, the precise mechanism by which direct or indirect binding of Rpl22p to the intronic regulatory element inhibits splicing remains to be investigated. We could not demonstrate that the regulatory element was sufficient to confer regulation *in vivo* when transposed into a different intron. However, the regulatory element was sufficient to sequester Rpl22p and therefore enhance *RPL22B* pre-mRNA splicing *in vitro* when added *in trans*. These results suggest that the regulatory element may not fold properly within the context of a foreign intron, but is functional when introduced in isolation, at least in *vitro*.

An Rpl22 autoregulatory system has previously been identified in higher eukaryotes. Based on previous knowledge that human Rpl22 binds strongly to Epstein-Barr virus-expressed RNA 1 (EBER1), an *in vitro* search for Rpl22 RNA ligands revealed a consensus motif characterized by an RNA stem closed with a G-C base pair that leads into a hairpin loop consisting of between 5 to 9 nucleotides, of which the 3’-most nucleotide is a uridine [36]. Accordingly, a recent study by O’Leary and colleagues identified an RNA-binding motif matching these characteristics within exon 2 of the zebrafish *Rpl22l1* mRNA that allows Rpl22 protein to bind and regulate the expression of the mRNA. They further confirmed that this binding is lost upon disruption of the hairpin loop [13]. In the yeast system, however, the regulatory element is entirely intronic and the fidelity of binding appears to be conferred by a combination of sequence-independent secondary structures and sequence-specific elements within the stem region. Specifically, our mutational analyses showed that the identity of the nucleotides within the apical hairpin loop of the *RPL22B* regulatory element is inconsequential in determining binding specificity (S1 Text and S4 Fig). It is also uncertain whether there is mechanistic overlap with the mammalian and zebrafish autoregulatory systems, specifically whether these mechanisms operate through inhibition of splicing as we have demonstrated to be the case in yeast. Ostensibly, however, the yeast and metazoan Rpl22 regulatory mechanisms appear to have the identical purpose of repressing one of the two Rpl22 paralogs. It remains to be seen whether the propensity to down-regulate Rpl22 paralogues has resulted from evolutionary conservation among eukaryotes, or if autoregulatory mechanisms have emerged as a result of convergent or parallel evolution.

### Why regulate *RPL22B*?

Although autoregulatory mechanisms exist for multiple ribosomal protein genes in yeast, it is not immediately clear what purpose they serve, if any, with regards to cell’s ability to respond to environmental stresses. A previous study found that ribosomal reprogramming and enrichment with the Rpl22Ap paralog occurs in yeast following exposure to oxidative stress, though it remains uncertain how this reprogramming ultimately affects the proteome [18]. Here we uncovered a role for the Rpl22 autoregulatory system in preventing the splicing of *RPL22B* pre-mRNA in conditions of MMS-induced DNA damage (Fig 5). At this juncture, we have not determined whether this inhibition is a direct effect of MMS treatment or is instead a downstream result of the DNA damage response. However, given that ribosome assembly is typically disrupted during DNA damage [50,51], it is possible that the MMS treatment triggers increased levels of free or complexed Rpl22p that are able to inhibit *RPL22B* splicing, perhaps similar to cadmium treatment (Figs 5D and S12). Because the overexpression of *RPL22B* cDNA did not result in any noticeable differences in cellular fitness compared to the wild-type cells when grown in a range of MMS concentrations (S11 Fig), we propose that, at least in yeast, the regulation of this specific protein is not essential to maximize cell survival in these conditions. Rather, it is more likely part of a broader gene repression program designed to respond to DNA damage, and is perhaps a result of the documented role of the spliceosome in promoting decay of the *BDF2* mRNA during DNA damage response [52]. Additionally, the availability of spliceosomal components is a key limiting factor in determining the efficiency of splicing; pre-mRNA transcripts “compete” for spliceosome components in situations when there is limited spliceosome available [53]. In this regard, the Rpl22 autoregulatory mechanism may help prevent the sequestration of the spliceosomal machinery on a non-essential transcript, leaving more spliceosomes available for the processing of more crucial transcripts, which may prove vital in cases of cellular stress. The additional observation that *RPL22B* down-regulation during rapamycin treatment does not appear to involve inhibition of splicing (Fig 5C) may further suggest that the mechanism features more prominently under specific stress conditions than in others, the reasons for which will be interesting inquiries for future experiments.

In mammalian cells Rpl22 has been shown to play a critical role in the maturation of αβ lineage T cell development as well as in early B cell development, both of which are dependent upon the induction of the p53 tumor suppressor [54,55]. Ablation of Rpl22 results in activation of p53 and the developmental arrest of both cell types in a tissue-specific manner. Interestingly, p53 is also activated in response to DNA damage, one of the early hallmarks of tumorigenesis [56]. While it remains to be determined whether Rpl22 expression is affected by DNA damage in mammalian cells as it is in yeast, it is tempting to speculate that the ability for yeast cells to down-regulate *RPL22B* during MMS-induced DNA damage may be a functional progenitor that evolved to modulate the induction of p53 in response to DNA damage in higher eukaryotes.

In summary, our study reveals an additional splicing-based autoregulatory pathway for a specific yeast RPG. In light of recent genome-wide analyses revealing numerous cases of negative feedback of RNA expression that serves to buffer allelic variants in yeast [57], we propose that additional mechanisms are in place to assist the cell with genetic autoregulation at many of these loci. Additional studies will delve into these mechanisms and help to demystify the intricacies of eukaryotic gene expression control.

## Materials and Methods

### Strain construction

Unless otherwise indicated, all mutant strains in this study were derived from the BY4742 background (S1 Table) or obtained directly from the GE Dharmacon Yeast Knockout Collection. Deletion mutants created in this study were constructed by transformation of the background strain with PCR products containing an auxotrophic marker or antibiotic resistance cassette as well as sufficient flanking sequences for efficient homologous recombination into the ORF of interest (see below). Successful transformants were screened by growth on dropout media or antibiotic plates and confirmed by PCR. Non-deletion chromosomal mutations including the insertion of the Myc tag into the chromosomal *RPL22A* locus, replacement of the *RPL18B* intron with the *RPL22B* intron, and deletion of the regulatory element from the *RPL22B* intron were achieved using the *delitto perfetto* approach [32]. Briefly, the CORE cassette was amplified from the pCORE plasmid by high-fidelity PCR (New England Biolabs No. M0530) using primer sets containing homologous sequences for the regions named above. CORE cassettes were transformed into yeast strains using the LiAc/SS carrier DNA/PEG approach [58]. Transformants were plated onto YPD and then replica plated one day later onto G418 plates. G418-resistant colonies indicating successful CORE integration were confirmed by PCR using flanking and internal primers. Next, for the Myc insertion and intron replacement mutations, high-fidelity PCR products were generated for the 13x Myc tag or the *RPL22B* intron containing flanking regions at the *RPL22A* or *RPL18B* ORFs, respectively. A total volume of 400 μl of PCR products was added to Eppendorf tubes containing 1 ml 100% EtOH and 40 μl 3 sodium acetate pH 5.2, precipitated for ≥30 minutes at -80°C, then centrifuged for 15 minutes at 15000 rpm to pellet the DNA. Pellets were washed with 500 μl 70% EtOH, centrifuged for 5 minutes, air dried and resuspended in 34 μl nuclease-free water. For the chromosomal deletion of the regulatory element from the *RPL22B* intron, overlapping oligonucleotides were used containing the deletion of interest and flanking regions for the *RPL22B* intron. See S1 Table for a full list of oligonucleotides used for these procedures. For all mutations, DNA was transformed into CORE-containing strains as described above. Transformants were plated on YPD and then replica plated onto 5-FOA plates one day later to screen for successful CORE excision. Colonies growing on 5-FOA were tested for G418 sensitivity to confirm CORE excision. Finally, genomic DNA was extracted from screened clones (see below) and mutations were confirmed by PCR and Sanger sequencing (Laragen, Inc). The *rpl31a*Δ and *rpl39*Δ strains were generously provided by Brian Kennedy (Buck Institute). The HA-tagged Prp42p strains were generously provided by Tracy Johnson (UCLA).

### Yeast cell culturing

For experiments involving untransformed cells, cultures were grown in rich media (YPD: 1% w/v yeast extract, 2% w/v peptone, and 2% w/v dextrose). Cells transformed with pUG23 or its derivative plasmids were grown in–HIS-MET synthetic dropout media (0.67% w/v yeast nitrogen base, 2% w/v dextrose, and 0.2% w/v powdered amino acid mixture without histidine and methionine). Cells transformed with pUG35, YEp24 or their derivatives were grown in–URA (for YEp24) or–URA-MET (for pUG35) synthetic dropout media (0.67% w/v yeast nitrogen base, 2% w/v dextrose, and 0.2% w/v powdered amino acid mixture without uracil or without uracil and methionine). For steady-state analysis experiments, cell cultures were grown to exponential phase until an OD_600_ of ~0.4–0.6 was attained. Cultures were then harvested in 50 ml volumes and centrifuged for 3 minutes at 3000 rpm (Sigma Rotor 11030), washed with deionized water and transferred to 2 ml screw-cap Eppendorf tubes. Cells were then centrifuged for 30 seconds at 13200 rpm in a benchtop microcentrifuge. Following removal of the supernatant, cells were flash frozen in liquid nitrogen. For MMS treatment experiments, 100% methyl methanesulfonate (Acros Organics No. AC15689-0050) was added to exponentially-growing cells to a final concentration of 0.05%. Cultures were then grown for an additional 90–180 minutes with culture harvests at regular intervals as indicated in the text. For heat shift experiments, exponentially-growing cells were centrifuged as described above and then sterile transferred to pre-warmed media at 37°C and grown for an additional three hours. Cultures were then harvested as described above. For rapamycin treatment experiments, 1 mg/ml rapamycin in 90% ethanol, 10% Tween 20 was added to a final concentration of 200 ng/ml. Cultures were then grown for an additional 30 minutes with harvests at the time points indicated in the text. For cadmium treatment experiments, cadmium chloride was added to exponentially-growing cells to a final concentration of 50 μM. Cultures were then grown for an additional 120 minutes with cultures harvests at 60 and 120 minutes as indicated in the text. See S1 Table for a full list of strains and plasmids used in this study.

### Yeast genomic DNA extraction

Freshly-streaked yeast cells were collected from media plates and added a 2 ml screw-cap Eppendorf tubes with 400 μl of genomic DNA lysis buffer (2% Triton X-100, 1% SDS, 100 mM NaCl, 10 mM Tris-HCl pH 8.0, 1 mM EDTA pH 8.0), 400 μl of acid-washed glass beads (Sigma No. G8772), and 400 μl of DNA Phenol-Chloroform (25 phenol:24 chloroform: 1 isomyl alcohol, pH 8.0, OmniPur). Samples were vortexed for 3 minutes at maximum intensity and then centrifuged for 5 minutes at 13200 rpm in a benchtop microcentrifuge. The top aqueous phase was transferred to a 1.5 ml microcentrifuge tube containing 400 μl of DNA phenol-chloroform. Samples were vortexed for 1 minute at room temperature and centrifuged at 15000 rpm for 5 minutes. 400 μl of the top aqueous phase were transferred to a 1.5 ml microcentrifuge tube containing 280 μl of 100% isopropanol. DNA was precipitated at -80°C for 30 min. The tubes were then centrifuged for 15 min at 15 krpm to pellet the DNA. After removing the supernatant, DNA pellets were washed with 500 μl of 70% ethanol, centrifuged for 5 min at 15 krpm, and resuspended in 300 μl of nuclease-free water. 1.5 μl of isolated gDNA was used for each 50 μL PCR reaction.

### RNA extraction

Cells were placed in 2 ml screw-cap Eppendorf tubes and frozen at -80°C. 350 μl of RNA buffer (50mM Tris-HCl pH 7.5, 100 mM NaCl, 10 mM EDTA pH 8.0), 350 μl of RNA buffer + 10% SDS, 700 μl of RNA Phenol-Chloroform (25 phenol:24 chloroform: 1 isomyl alcohol, pH 6.7, OmniPur), and 400 μl of acid-washed glass beads were added to cells. Afterwards, cells were vortexed at 3°C for 1 min, with 2 min intervals on ice for a total of 3 cycles. The tubes were then incubated for 6 minutes at 65°C. After incubation, tubes were vortexed for 1 min at high speed and centrifuged for 5 min at 13.2 krpm. 600 μl of the top aqueous layer was transferred to a 1.5 ml microcentrifuge tube containing 600 μl of RNA phenol-choloroform. Samples were vortexed for 1 minute at room temperature and centrifuged at 15krpm for 2 minutes. 500 μl of the top aqueous phase was transferred to a 1.5 ml microcentrifuge tube containing 500 μl of RNA phenol-chloroform. Samples were vortexed for 1 minute at room temperature and centrifuged at 15krpm for 2 minutes. Finally, 400 μl of the top aqueous phase were transferred to a 1.5 ml microcentrifuge tube containing 1 ml of 100% ethanol and 40 μl of 3M sodium acetate pH 5.2. Tubes were then cooled at -80°C for 30 min. The tubes were then centrifuged for 15 min at 15 krpm to pellet the RNA. After removing the supernatant, RNA pellets were washed with 500 μl of 70% ethanol, centrifuged for 5 min at 15 krpm, and resuspended in 30–40 μl nuclease-free water.

### Preparation of splicing extracts

To prepare splicing extracts, 3 liters of cell culture were grown to OD_600_ ≈ 1.6 as described above and harvested in 500 ml volumes by centrifugation at 5000 RPM for 10 minutes in a Beckman Coulter Avanti J-20 XP centrifuge with a JLA-8.1 rotor chilled to 4°C. The supernatant was removed and cell pellets were resuspended in 400 ml of ice cold buffer AGK (10 mM HEPES pH 7.9, 1.5 mM MgCl_2_, 200 mM KCl, 10% glycerol, 0.5 mM DTT) and centrifuged again for 10 minutes at 5000 RPM. The supernatant was removed and the cells were resuspended in 30 ml of cold AGK and transferred to a 50 ml centrifuge tube. The cells were centrifuged for 10 minutes at 3500 RPM in a Beckman Coulter Avanti J-25 centrifuge with a JA-25.5 rotor chilled to 4°C. The supernatant was removed and the cells were resuspended in a 0.4 volume of buffer AGK supplemented with 1x protease inhibitor cocktail (Roche No. 04693132001). Cells were then flash frozen via drip suspension into liquid nitrogen and stored at -80°C. For lysis, frozen cells were added to a 50 ml grinding jar (Retsch No. 014620216) containing a 25 mm grinding ball (Retsch No. 053680105) that were pre-chilled in liquid nitrogen. Cells were milled in a Retsch MM400 mixer mill for 5 alternating 3 minute periods of 11 and 12 Hz. Powdered lysate was transferred to an oakridge centrifuge tube and stored at -80°C. To prepare the extract, the powdered lysate was thawed on ice and centrifuged for 30 minutes at 17000 RPM at 4°C. (Beckman Coulter Avanti J-25 centrifuge with JA-25.5 rotor). The supernatant was then transferred to an ultracentrifuge tube and centrifuged at 37000 RPM for 1 hour at 4°C (Beckman Coulter Optima LE-80K Ultracentrifuge with Type 70.1 Ti rotor). Following ultracentrifugation, the middle aqueous phase was extracted and dialyzed in 3.5K MWCO Slide-A-Lyzer dialysis cassettes (Life Technologies No. 66332) in dialysis buffer (20 mM HEPES, 0.2 mM EDTA, 50 mM KCl, 20% glycerol, 0.5 mM DTT, 0.1 mM PMSF) under gentle agitation for two hours, after which the buffer was replaced and dialysis was performed for another 2 hours. Dialyzed splicing extract was transferred from the cassette to microcentrifuge tubes and centrifuged at 10 krpm for 1 minute at 4°C. The supernatant was then aliquoted into to fresh microcentrifuge tubes, frozen in liquid nitrogen, and stored at -80°C until needed.

### *In vitro* splicing assays

Pre-mRNA templates were transcribed using the MEGAscript T3 Transcription Kit according to the manufacturer’s instructions (Thermo Fisher No. AM1338). *In vitro* splicing reactions were assembled in 25 μl volumes, each containing 10 μl of splicing extract, 5 μl of 5x splicing buffer (12.5 mM MgCl_2_, 15% PEG 8000, 300 mM KH_2_PO_4_ at pH 7.2, and 30 μl nuclease-free water), 5 μl pre-mRNA template from *in vitro* transcription, 0.5 μl 200 mM ATP, 1 μl RNaseOUT Recombinant RNase Inhibitor (Life Technologies No. 10777–019), and 3.5 μl nuclease-free water. For reactions involving the addition of regulatory element RNA, this RNA was added to reactions in lieu of water to the final concentrations indicated in the figures. Reactions were incubated at 25°C for 40 minutes and then stopped by the addition of 75 μl nuclease-free water and 100 μl of RNA buffer followed immediately by phenol-chloroform RNA extraction. The extracted RNA was DNase-treated and then used for RT-PCR analysis (see below). Equal amounts of DNase-treated RNA were used for all samples for the cDNA synthesis reactions.

### Northern blotting

Total RNA aliquots (5–10 μg) were combined with glyoxal buffer to a volume/volume ratio of 5 parts glyoxal buffer to 1 part RNA solution. Glyoxal buffer consists of 60% Dimethyl sulfoxide (Sigma No. D8418), 20% deionized glyoxal (Sigma No. 50649), 1X BPTE [10 mM PIPES free acid (Sigma No. P6757) 30 mM Bis-Tris (Sigma No. B9754), 1 mM EDTA], 4.8% glycerol, and 40 μg/ml ethidium bromide). Glyoxylated RNA was heated for 1 hour at 55°C then cooled in an ice water bath for 10 min. Samples were then vortexed and centrifuged before loading onto agarose gels (between 1.5–2.2% agarose). Samples were separated via electrophoresis at 120V for 2–3 hours in 1X BPTE buffer with constant agitation by magnetic stir bars. Following separation, gels were photographed using a UV imager. Gels were then rinsed in deionized water for 10 minutes followed by a 20 minute equilibration in 75 mM NaOH and two additional 10 minute washes in deionized water. RNA was then blotted onto Amersham Hybond N^+^ membranes (VWR No. 95038) overnight with 10X SSPE used as the conducting buffer. 20x SSPE is 25 mM EDTA, 3 M NaCl, and 200 mM NaH_2_PO_4_-H_2_O. RNA was permanently crosslinked to the membrane using UV Stratalinker and hydrated with 2X SSPE. Membranes were pre-hybridized for 30–60 min at 65°C in 10–25 ml of Church’s buffer (1% w/v bovine serum albumin, 1 mM EDTA, 0.5 M NaPO_4_ pH 7.2, 7% SDS), after which 1–100 pmol of radiolabeled riboprobe was added to the buffer and hybridized for at least 8 hours (see below). Following hybridization, membranes were first washed at 65°C for 20 min with 2X SSPE, 0.1% SDS, followed by additional 20 minute washes at 65°C with 1X SSPE, 0.1% SDS, and 0.1X SSPE, 0.1% SDS. Depending on strength of signal following the washes, membranes were exposed in a Kodak K-screen between 15 minutes and 4 days and scanned with a Bio-Rad FX imager. Where indicated, band intensities were quantified using Quantity One volume analysis (Bio-Rad).

### DNase treatment, cDNA synthesis and RT-PCR analysis

DNase treatment was performed with Ambion Turbo DNase (Life Technologies No. AM2238). For each sample 40 μg of total RNA was aliquoted to 1.5 ml Eppendorf tubes and combined with 20 μl of 10x DNase Buffer, 4 μl of Turbo DNase, and nuclease-free to a final volume of 200 μl. Mixtures were incubated at 37°C to digest genomic DNA. Samples were then subject to RNA extraction as described above. For cDNA synthesis, 1–5 μg of DNase-treated RNA was combined with 0.4 μl dNTP mix (25 mM), 1 μl 50 ng/μl Random Hexamer (Life Technologies No. N8080127), and nuclease-free water to a final volume of 12 μl. Samples were then heated to 65°C for 5 minutes and chilled on ice for 2 minutes to anneal hexamers. Next, 4 μl 5x First Strand Buffer (250 mM Tris-HCl (pH 8.3), 375 mM KCl, 15 mM Magnesium Chloride), 1 μl RNaseOUT, and 1 μl M-MLV Reverse Transcriptase (Life Technologies No. 28025–021) were added to each sample. Samples were mixed gently and incubated at 25°C for 10 minutes, followed by a 50 minute incubation at 42°C and then a 5 minute incubation at 85°C to heat kill the enzymes. Between 1–1.5 μl of cDNA was used for 25 μl PCR reactions with Cy3-labeled primers under standard procedures (see S1 Table for list of primers). Prior to analysis, PCR reactions were mixed with equal volumes of denaturing loading dye (0.02% bromophenol blue, 20 mM EDTA in formamide solvent) and heated at 95°C for 10 minutes. Samples were then analyzed in a Sequi-Gen GT Nucleic Acid Electrophoresis Cell (Bio-Rad No. 165–3860) using 5% denaturing acrylamide gels [8 M urea, 5% Acrylamide:Bis-acrylamide 19:1 (Fisher No. BP1406-1), polymerized with 8.3% ammonium persulfate and 1.6% TEMED]. Samples were separated at 120W for 1–2 hours and gels were dried and analyzed in a Bio-Rad FX imager.

### RNA-Immunoprecipitation (RIP)

Prior to RIP, rabbit anti-Myc antibodies (Santa Cruz Biotechnology No. sc-789) were conjugated to Protein G Sepharose beads (GE Healthcare No. 17-0618-01). Briefly, 20 μl of beads per sample were aliquoted into a 1.5 ml Eppendorf tube and centrifuged for 15 seconds at 10000 rpm. The supernatant was removed and beads were washed twice with 500 μl of cold NET-2 buffer (40 mM Tris-HCl pH 7.5, 150 mM NaCl, 0.05% IGEPAL). 2 μg of antibody per sample was added to the beads and conjugated by gentle rotisserie rotation at 4°C for 1 hour. Beads were then washed three times with 500 μl cold NET-2 buffer to remove unbound antibody.

Yeast cell cultures were grown, harvested, pelleted and flash-frozen as described above, except using low-retention 1.5 ml Eppendorf tubes (Fisher No. 02-681-331). Pellets were resuspended in 600 μl NET-2 buffer and 400 μl acid-washed glass beads. Samples were vortexed at 4°C at maximum intensity for 45 seconds on and 45 seconds off for a total of six cycles. The supernatant was isolated by needle-puncturing the tube and centrifugation into clean 1.5 ml microcentrifuge tubes. Samples were then centrifuged at 4°C for 20 minutes to pellet any remaining cell debris. The resulting supernatant was used to calculate aliquot volumes for immunoprecipitation. Briefly, OD_260_ measurements were made using a Nanodrop 2000 spectrophotometer (Thermo Scientific) to determine O.D_260_ units per ml of sample. Sufficient supernatant aliquots for 2.5 OD_260_ units were added to 20 μl antibody-conjugated Protein G Sepharose beads and brought to a final volume of 400 μl with NET-2 buffer. For the RIP samples, RNA was immunoprecipitated by gentle rotisserie rotation at 4°C for 1 hour. Following IP, samples were washed six times with 1 ml cold NET-2 buffer. RNA was then extracted and prepared for RT-PCR analysis as described above. For the input samples, supernatant aliquots for 2.5 OD_260_ were added to safe-lock Eppendorf tubes. RNA was then extracted and prepared for RT-PCR analysis as described above.

### Riboprobe synthesis

Riboprobes were prepared in a 20 μl reaction using Promega T3 RNA Polymerase (Promega No. P4024) according the manufacturer’s protocol utilizing DNA templates containing T3 promoter sequences. See S1 Table for list of oligonucleotide primers used to generate T3 templates. Following riboprobe synthesis, the reaction mixture was brought to a total volume of 200 μl with nuclease-free water. The riboprobe solution was then added to the pre-hybridized membranes as described above.

### Oligoprobe radiolabeling

Oligonucleotides were labeled with γ-P^32^ ATP using T4 polynucleotide kinase (New England Biolabs No. M0201). Reactions were prepared to a 10 μL volume consisting of 3 μL deionized nuclease-free water, 1 μL 10x polynucleotide kinase buffer (NEB No. B0201), 2 μL 10 mM oligonucleotide solution, 3 μL γ-P^32^ ATP at 10 μCi/μl, and 1μl T4 polynucleotide kinase enzyme. Reactions were incubated at 37°C for 45 minutes and then brought to a final volume of 30 μl with deionized nuclease-free water. The oligonucleotide solution was then added to the pre-hybridized membrane and hybridized at 42°C for at least 8 hours. Washing steps were similar to those described in Northern Blotting procedures except that they were conducted at 42°C and excluded a wash with 0.1x SSPE, 0.1% SDS.

### Cloning and bacterial transformation

To clone the *RPL22B* intron into pUG35, the intron was amplified by PCR from genomic DNA using primers that insert BamHI (NEB No. R3136) and EcoRI (NEB No. R0101) restriction sites into the ends of the PCR product. PCR reactions were performed using Phusion Hi-Fi Polymerase (NEB No. M0530) according to the manufacturer’s protocol. PCR product was purified using a microcentrifuge spin column according to the manufacturer’s protocol (BioPioneer No. PPP-100). Amplification product and pUG35 vector were digested using BamHI and EcoRI restriction enzymes for 2–16 hours. Restriction digest was done using 1 μl 100X BSA, 10 μl NEBuffer, 1 μl CIP (NEB No. M0290, for vector only) and water. Ligation of insert to vector was then performed using T4 DNA Ligase (Life Technologies No. 15224) according the manufacturer’s protocol. Plasmids were transformed into DH5α competent E.coli cells (see below) and plated on LB Agar plates with 100 μg/ml ampicillin. Screening of colonies for positive clones was done by growing liquid cultures for miniprep (BioPioneer No. CMIP-100) followed by PCR (see S1 Table for primers used) and positive clones were confirmed by sequencing (Laragen Inc.).

To clone intronless *RPL22A* cDNA into pUG35, total RNA from wild-type cells was reverse-transcribed as described above (see cDNA synthesis). The short length of *RPL22A* exon 1 allowed for the use of a forward primer specific for the spliced cDNA. Forward and reverse primers inserted the BamHI and EcoRI restriction sites into the ends of the PCR product, which was then purified and cloned into pUG35 as described above. Cloning of intronless *RPL22A* cDNA into YEp24 was performed in a similar manner except the reverse primer incorporated the EagI restriction site to the end of the PCR product, which was then purified and cloned into YEp24 as described above.

### Site-directed mutagenesis of plasmid DNA

Site-directed mutagenesis was performed using the QuikChange Lightning Site-Directed Mutagenesis Kit (Agilent Technologies No. 210518). Each reaction combined 5 μl of 10X reaction buffer, 10–100 ng of dsDNA template, 125 ng of oligonucleotide primer #1, 125 ng of oligonucleotide primer #2, 1 μl of dNTP mix, 1.5 μl of QuikSolution reagent, 1 μl of QuikChange Lightning Enzyme and nuclease-free water to a final volume of 50 μl. Oligonucleotides were designed using the QuikChange Primer Design online tool. See S1 Table for a complete list of oligonucleotides used for mutagenesis. Mutagenesis reactions were performed in a thermocycler according to the manufacturer’s instructions. After the reaction cycle, each amplification reaction was mixed with 2 μl of DpnI restriction enzyme and then incubated at 37°C for 5 minutes to digest the parental dsDNA. DpnI-treated DNA was then transformed into XL10-Gold ultracompetent cells per the manufacturer’s instructions which were then plated onto LB + Ampicillin plates and incubated at 37°C for 16 hours.

### DNA transformations into bacteria and yeast

Aliquots of DH5α competent E.coli were added to 1.5 ml Eppendorf tubes and plasmids were added in a 10% v/v amount. Tubes were then incubated on ice for 10 minutes, subject to heat shock for 30 sec at 42°C, and then incubated on ice for 5 minutes. The transformation mixture was resuspended in 1 ml of SOC media (20 mM MgSO_4_, 2.5 mM KCl, 10 mM NaCl, 2% bacto tryptone, 0.5% yeast extract, 20 mM glucose) and incubated for 1 hour at 37°C. Tubes were then centrifuged for 1 min at 10krpm. After removing most of the supernatant, cell pellets were resuspended in the remaining supernatant (~100 μl) and plated onto LB Agar plates with 100 μg/ml ampicillin. Glass beads were then added to spread cells onto plates until all liquid was absorbed by the plate media. Plated E. coli were then incubated at 37°C. Yeast plasmid and linear DNA transformations were conducted using the LiAc/SS carrier DNA/PEG method [58].

### Spot dilution assays

Agar plates for the spot dilution assays were prepared similarly to the–URA and–HIS-MET liquid media described above, except with the addition of 2% w/v agar. For the testing of cell growth in MMS, media was cooled to ~65°C following sterilization after which 100% MMS (Acros Organics No. 200001–324) was added to the media to a final concentration of 0.005%, 0.01%, or 0.03%.

For the antibiotic screen media was cooled to ~65°C following sterilization after which antibiotics were added to the following concentrations: 0.5 μg/ml anisomycin, 200 μg/ml paromomycin, 100 μg/ml puromycin, 4 mM neomycin, and 2 μg/ml verrucarin A. After the antibiotics or MMS were fully dissolved and mixed, the media plates were poured under sterile conditions and allowed to solidify overnight. Plates were used within two days after preparation. Neomycin, anisomycin, puromycin and verrucarin A were generously provided by Q. Al-Hadid and S. Clarke (UCLA). Paromomycin sulfate was purchased from VWR (Cat. #AAJ61274-06).

Yeast cell cultures were grown in–URA liquid media to exponential phase until an OD_600_ of ~0.4–0.6 was attained. 1 ml volumes of cell culture were then transferred to 1.5 ml Eppendorf tubes and cells were collected by spinning in a microcentrifuge for 30 seconds at 13200 rpm. Cell pellets were resuspended in 1 ml of sterile water and further diluted to OD_600_ = 0.10 in a fresh 1.5 ml Eppendorf tube with sterile water. Cells were then subject to five 5-fold series dilutions using sterile water in clean 1.5 ml Eppendorf tubes. For each dilution 4 μl of cell suspension was spotted onto the media plates presented in S10 and S11 Figs. Plates were incubated at 30°C for 1.5–3 days.

### Western blot analysis

Cell cultures were grown to exponential phase in rich media (YPD, see above). Cultures were then harvested in 50 ml volumes and centrifuged for 3 minutes at 3000 rpm (Sigma Rotor 11030), washed with deionized water and transferred to 1.5 ml low-retention Eppendorf tubes (Fisher No. 02-681-331). Cells were then pelleted by centrifugation for 30 seconds at 13200 rpm in a benchtop microcentrifuge. Following removal of the supernatant, cells were flash frozen in liquid nitrogen. To lyse the cell pellets, 200 μl of high-salt lysis buffer was added to each sample [200 mM Tris-HCl pH 8.0, 320 mM Ammonium Sulfate, 5 mM MgCl_2_, 10 mM EGTA pH 8.0, 20 mM EDTA pH 8.0, 1 mM DTT, 20% glycerol, 1 mM PMSF, 20 mM Benzamidine HCl, and 1x Protease Inhibitor cocktail] along with 200 μl acid-washed glass beads. Samples were vortexed at maximum intensity for 8 minutes at 4°C. Cellular debris was pelleted by centrifugation at 3000 rpm for 3 minutes and the supernatant was transferred to clean 1.5 ml Eppendorf tubes. Samples were then centrifuged again at 15000 rpm for 10 minutes at 4°C and the supernatant was transferred to clean 1.5 ml Eppendorf tubes. Lysate protein concentration was quantified with Bio-Rad Bradford protein assay (Bio-Rad No. 500–0001).

To prepare protein electrophoresis samples, 10 μg of protein and water were combined to a total volume of 15 μl, which was then brought to a total volume of 20 μl with the addition of 5 μl 20% β-mercaptoethanol in 4x loading dye. Samples were boiled for 5 minutes and then loaded into 10% SDS-PAGE gels (10% resolving gel composed of 375 mM Tris-HCl pH 8.8, 8% acrylamide, 0.1% SDS, 0.1% APS, and 0.1% TEMED, and 3% stacking gel composed of 126 mM Tris-HCl pH 6.8, 3% acrylamide, 0.1% SDS, 0.1% APS, and 0.1% TEMED). Samples were run for ~1 hour at 180 V. Following electrophoresis, protein was transferred to a 0.22 μm PVDF membrane in 1x Transfer Buffer (20% methanol, 25 mM Tris Base, 192 mM glycine) for 1 hour at 100 V. Protein transfer was confirmed by Coomassie blue staining. Following de-stain, the membrane was incubated in 5% blocking buffer [5% w/v powdered milk in 1x PBS-T (10 mM Sodium Phosphate, 150 mM NaCl, 0.05% Tween 20)] for 1.5 hours at room temperature. Following blocking, the membrane was washed once with 1x PBS-T for 10 minutes. Next, the membrane was incubated with the primary rabbit anti-Myc antibody (Santa Cruz Biotechnology No. sc-789) at a 1:5000 dilution in 5% blocking buffer overnight at 4°C. The membrane was then washed three times for 10 minutes each in 1x PBS-T, and then incubated with the secondary goat anti-rabbit antibody (Pierce No. 31460) at a 1:10000 dilution for one hour at room temperature. The membrane was washed four times for 15 minutes each in 1x PBS-T. To visualize the bands, the membrane was incubated with SuperSignal West Femto Chemiluminescent Substrate (Pierce No. 34096) for 5 minutes and then exposed to x-ray film.

### Chromatin immunoprecipitation (ChIP)

Yeast cells expressing C-terminal HA-tagged Prp42p and carrying YEp24 control of *RPL22A* overexpression plasmids were grown in 150 mL cultures to exponential phase at 30°C as described above, after which 37% formaldehyde was added to a final concentration of 1% to initiate crosslinking. Cultures were then incubated with shaking at room temperature for an additional 15 minutes, followed by the addition of glycine to a final concentration of 125 mM and another 5 minute incubation at room temperature to quench the crosslinking. Cells were then pelleted by centrifugation as described above, washed twice in 25 mL of ice cold 1x PBS buffer, collected and flash frozen in liquid nitrogen. The cell pellets were then thawed in 1 mL Lysis Buffer A (50 mM HEPES-KOH pH 7.5, 140 mM NaCl, 1 mM EDTA, 1% Triton X-100, 0.1% sodium deoxycholate, 1x protease inhibitor cocktail, 1 mM PMSF) and lysed with the addition of 300 μL acid-washed glass beads and vortexing for 40 minutes at 4°C. The supernatant was then collected into clean 1.5 mL microcentrifuge tubes. Next, the chromatin was subject to shearing by sonication at maximum intensity at 4°C for 7 cycles of 30 seconds on and 1 minute off. The sonicated chromatin was centrifuged for 10 minutes at 4000 RPM and the supernatant was transferred to a fresh 2 mL microcentrifuge tube. 20 μL of cleared chromatin was set aside and flash frozen for the input sample, and 700 μL of chromatin was transferred to a 1.5 mL microcentrifuge tube and incubated at 4°C with 2 μg of rabbit anti-HA primary antibody (Santa Cruz Biotechnology No. Y-11) for 4 hours with gentle rotation. Next, 40 μL of 50% slurry of ImmunoPure Protein A beads (Pierce No. 20333) were added to the chromatin/antibody mixture and the samples were incubated overnight at 4°C with gentle rotation. Following incubation, the samples were briefly spun to pellet the beads and the supernatant was removed. The beads were then subject to the following washes: three washes with 500 μL Lysis Buffer A, one wash with 500 μL Lysis Buffer B (50 mM HEPES-KOH pH 7.5, 500 mM NaCl, 1 mM EDTA, 1% Triton X-100, 0.1% sodium deoxycholate, 0.1% SDS, 1x protease inhibitor cocktail, 1 mM PMSF), one wash with 500 μL lithium chloride buffer (10 mM Tris-HCl pH 8, 250 mM LiCl, 5 mM EDTA, 1% Triton X-100, 0.5% NP-40, 1x protease inhibitor cocktail, 1 mM PMSF), and one wash with 1 mL 1x TE buffer (100 mM Tris-HCl pH 8, 10 mM EDTA). The supernatant was removed and 250 μL of elution buffer (50 mM Tris-HCl pH 8, 5 mM EDTA, 1% SDS) was added to the beads. 200 μL elution buffer was also added to the input samples that had been thawed on ice. The input and ChIP samples were incubated overnight at 65°C to reverse the crosslinks and then spun briefly to pellet the beads. The supernatant was transferred to fresh 1.5 mL microcentrifuge tubes and treated with 4 μL of 10 mg/mL proteinase K for 1 hour at 42°C. The sample was then cleaned using the QIAquick PCR Purification Kit (Qiagen No. 28104) and eluted into 50 μL of sterile water. Finally, the eluted chromatin was treated with 1 μL of 10 mg/mL RNase A at 37°C for 1 hour and used for PCR analysis.

## Supporting Information

S1 Fig **A.** *Left*: Comparative table for *RPL22A* and *RPL22B*. Assembled ribosome proportions and percent nucleotide and amino acid identities are provided. *Right*: Scale diagrams comparing *RPL22A* (upper) and *RPL22B* (lower) pre-mRNAs, with the exonic sequences in grey and the introns represented by black lines. The nucleotide positions are labeled with respect to the first nucleotide of the coding sequences. The 5’UTR (blue), alternative 5’-splice site, regulatory element, and RT-PCR primer positions are shown for *RPL22B*. **B.** Northern blot detecting the splicing of *RPL22B* pre-mRNA in wild-type and *upf1*Δ mutant strains carrying the empty pUG23 vector or the *RPL22B* overexpression plasmid. Labeled bands show the unspliced (US) and spliced (S) species. Transcripts were detected using an *RPL22B* 5’UTR riboprobe. *SCR1* was used as a loading control. **C.** Northern blot detecting the splicing of *RPL22A* pre-mRNA in wild-type and *upf1*Δ mutant strains carrying the empty pUG23 vector or the *RPL22A* overexpression plasmid. Transcripts are labeled similarly to panel A and were detected using an *RPL22A* 3’UTR riboprobe. *SCR1* was used as a loading control. **D.** Northern blot detecting the splicing of *RPL22A* pre-mRNA in wild-type and *upf1*Δ mutant strains carrying the empty pUG23 vector or the *RPL22B* overexpression plasmid. Detection methods and labels are similar to panel C. **E.** Northern blot detecting the splicing of *RPL22A* pre-mRNA in wild-type, *upf1*Δ, *rpl22b*Δ, and *upf1*Δ*rpl22b*Δ mutant strains. Detection methods and labels are similar to panel C.
(TIF)

S2 Fig Systematic search for the *RPL22B* intron regulatory element. **A.** Northern blot detecting splicing of the *RPL22B* intron reporter transcript expressed from constructs with the full intron or deletion of intronic bases 7 though 152 (Δ7–152) or 153 through 297 (Δ153–297) in strains with or without the chromosomal deletion of *RPL22A*. Labeled bands show the unspliced (US), alternatively-spliced (*AS 5’), and spliced (S) species. Transcripts were detected using a *GFP* ORF riboprobe. *SCR1* was used as a loading control. **B.** RT-PCR analysis of the *RPL22B* intron reporter transcript expressed from constructs with full intron, Δ7–152, or Δ153–297 in strains with or without the chromosomal deletion of *RPL22A*. **C.** Northern blot detecting splicing of the *RPL22B* intron reporter transcript expressed from constructs with the full intron, Δ7–152, or Δ153–297 in strains with or without the chromosomal deletion of *RPL22A* and with or without the deletion of the *RPL22B* alternative 5’ splice site. Bands are labeled similarly to panel B. Ethidium bromide-stained 18S rRNA is shown as a loading control.
(TIF)

S3 Fig Conservation analysis of the *RPL22B* intron. **A.** The full regulatory element as predicted by Mfold with key nucleotides labeled for reference. Highly conserved nucleotides include both the upstream and downstream nucleotides and are denoted with the black bar. Nucleotides G-153, U-183, A-220 and G-246 have been labeled in colors corresponding to the boxes in the accompanying conservation plots in panel B. **B.** UCSC genome browser snapshots showing strong sequence conservation of intronic nucleotides forming the lower regions of the regulatory element among yeast. *Top panel*: Snapshot of the entire intron. *Center panel*: Snapshot showing conservation of the upstream nucleotides constituting the bottom half of the stem loop, corresponding to intronic nucleotides 153 through 183. *Bottom panel*: Snapshot showing conservation of the downstream nucleotides constituting the bottom half of the stem loop, corresponding to intronic nucleotides 220 through 246. Note that these plots provide the sequence of the Watson strand while *RPL22B* is encoded on the Crick strand. **C.** Structure of the regulatory element as predicted by Mfold when intronic bases 226 through 297 are removed. The nucleotides in green have been determined to promote the regulation of splicing (see text and S5 Fig).
(TIF)

S4 Fig Targeted mutational analysis of the regulatory element. **A.** Predicted Mfold structure of the distal end of the Full regulatory element showing the upper and lower distal stems, the hairpin loop, and the intervening RNA loop between the two distal stems. **B.** Northern blot detecting the splicing of the *RPL22B* intron reporter transcript expressed from constructs with full intron, Δ153–188, or deletion of intronic bases 181 through 191 (Δ181–191) or 212 through 223 (Δ212–223) in wild-type cells. Bands are labeled similarly to panels D-E. Transcripts were detecting using a GFP ORF riboprobe. *GAPDH* was used as a loading control. **C.** Predicted Mfold structure of the distal end of the modified construct Δ191–211 UUCG showing the ultrastable tetraloop. **D.** Northern blot detecting the splicing of the *RPL22B* intron reporter transcript expressed from constructs with full intron, Δ181–191, and the Δ191–211 UUCG construct in wild-type cells. Bands are labeled similarly to panels D-F. *SCR1* was used as a loading control. **E.** Predicted Mfold structures of the “Flip” and “Scramble” constructs, respectively showing the inversion and reordering of the nucleotides of the lower distal stem. **F.** Northern blot detecting the splicing of the *RPL22B* intron reporter transcript expressed from constructs with full intron, Δ181–191, Δ191–211 UUCG, or Δ191–211 UUCG with the lower distal stem bases flipped (Δ191–211 UUCG “Flip”) or scrambled (Δ191–2211 UUCG “Scramble”) in wild-type cells. Bands are labeled similarly to panels B and D. *SCR1* was used as a loading control.
(TIF)

S5 Fig Additional targeted mutational analysis of the regulatory element. **A.** Predicted Mfold structures of constructs tested in panels B and C. Nucleotides of interest are colored red. **B.** Northern blot analysis of *RPL22B* intron reporter transcript expressed from constructs with full intron, Δ181–191, Δ191–211 UUCG, Δ191–211 UUCG with the deletion of the RNA internal loop (Δ191–211 UUCG Δinternal loop), deletion of the lower distal stem and intervening internal loop (Δlower distal stem), mutation of the upstream RNA internal loop sequence from CCCU to AAAC (US Internal Loop AAAC), or mutation of the downstream RNA internal loop sequence from UGAA to CAUU (DS Internal Loop CAUU) in wild-type cells. Labeled bands indicate the unspliced (US) and spliced (S) species. Bands were detected using a GFP ORF riboprobe. *SCR1* was used as a loading control. **C.** Northern blot analysis of *RPL22B* intron reporter transcript expressed from constructs with full intron, Δ181–191, US Internal Loop AAAC, DS Internal Loop CAUU, or mutation of the upstream RNA internal loop sequence from CCCU to UUCA (US Internal Loop UUCA). Bands are labeled similarly to panel A. *SCR1* was used as a loading control.
(TIF)

S6 Fig The deduced regulatory element is necessary but not sufficient to confer inhibition of splicing *in vivo*. A. Comparison of key intronic properties of *RPL22B* and *RPS21A*. B. Northern blots detecting the splicing patterns of *RPS21A* (upper panel) and *RPL22B* (lower panel) in strains with the empty YEp24 vector or the *RPL22A* overexpression plasmid. The strains carry either the wild-type *RPS21A* intron or mutated versions of the intron harboring either the *RPL22B* regulatory element (22B-reg) or the an 87 nucleotide intronic deletion (Δi87). *GAPDH* was used as a loading control. See S1 Text for additional details.
(TIF)

S7 Fig Full RT-PCR gel images of the regulatory element addition *in vitro* splicing experiment. These are non-attenuated images of the gels that are presented in truncated form in Fig 4E.
(TIF)

S8 Fig Western blot of tagged protein and full gel images of RIP experiments. **A.** Western blot confirmation of the Myc epitope tag on Rpl22Ap. The untagged strain was used as a negative control. Strains expressing Rpl22Ap with a single epitope tag and a Myc-tagged Smd2 protein were used as positive controls. Three separate clones of strains expressing 13-Myc tagged Rpl22Ap were each tested in WT background and the *RPL22B* intron regulatory element deletion background (*RPL22Bi reg*Δ). The Myc epitope tag was detected using an anti-Myc primary antibody. Coomassie blue stained proteins are shown as a loading control. **B.** Full unattenuated gel images that were presented in truncated form in Fig 3D.
(TIF)

S9 Fig Rpl22p and rRNA within the native eukaryotic ribosome. **A.** PyMol 3D views of the yeast Rpl22 protein and the proximal rRNA sequence. Clockwise from upper left: Rpl22p in the context of all large subunit rRNA, frontal view of Rpl22 and proximal rRNA secondary structure, left side view, and right side view. Protein and RNA structures are from [34]. **B.** 2D representation of the rRNA secondary structure from [35].
(TIF)

S10 Fig Growth of yeast overexpressing *RPL22* paralogs in the presence of ribosomal inhibitors. Growth of WT and *rpl22a*Δ strains carrying an empty YEp24 vector or *RPL22* paralog overexpression plasmids in various ribosome-inhibiting antibiotics. Following 5-fold spot dilution, plates were incubated at 30°C for the number of days indicated.
(TIF)

S11 Fig Splicing changes for *RPL22B* and growth of yeast overexpressing *RPL22* paralogs in the presence of MMS. A. Quantification of spliced *RPL22B* transcript in WT and *RPL22Bi reg*Δ strains after three hours of treatment with 0.05% MMS reported as a percentage of spliced transcript that was present prior to treatment (t = 0). The values shown are mean ± 1 standard deviation measured from Northern blot experiments consisting of three independent biological replicates for each strain. B. Quantification of unspliced and spliced *RPL22B* transcripts in wild-type, *RPL22Bi reg*Δ, and *rpl22a*Δ strains at steady state and following three hours of treatment with 0.05% MMS, given as percentage of the total transcript. The values shown are mean ± 1 standard deviation measured from Northern blot experiments consisting of three independent biological replicates for each strain. C. Wild-type cells carrying an empty YEp24 vector or plasmids overexpressing the intronless cDNA of *RPL22A* and *RPL22B* were tested for growth in 0%, 0.005%, 0.01% and 0.03% MMS. Following 5-fold spot dilution, plates were incubated at 30°C for the number of days indicated.
(TIF)

S12 Fig rRNA transcription and maturation is inhibited by cadmium chloride treatment. Northern blots detecting the 35S rRNA and other rRNA processing intermediates. Cells were grown in YPD containing 50 μM CdCl_2_ for 120 minutes. The 35S rRNA and processing intermediates were detected using oligoprobes complementary to transcripts arising from the *RDN1* locus as shown in the lower diagram. Sequences for O1660 and O1663 were obtained from Lindahl et al. [59]. *SCR1* was used as a loading control.
(TIF)

S13 Fig Full gel image of the *in vitro* splicing and RT-PCR experiment utilizing wild-type splicing extracts. This is a non-attenuated image of the gel that is presented in truncated form in Fig 4C.
(TIF)

S14 Fig Full gel image of the *in vitro* splicing and RT-PCR experiment utilizing wild-type and *RPL22A* overexpression splicing extracts. This is a non-attenuated image of the gel that is presented in truncated form in Fig 4D.
(TIF)

S1 Text Comprehensive description of experiments to elucidate the regulatory element. (DOCX)

S1 Table List of yeast strains, plasmids, and oligonucleotides used in this study. (XLSX)
