# Supplementary figures and images for: Splicing-Mediated Autoregulation Modulates Rpl22p Expression in Saccharomyces cerevisiae
Source: PLoS Genet. 2016 Apr 20;12(4):e1005999. doi: 10.1371/journal.pgen.1005999 (PMC4838235; doi:10.1371/journal.pgen.1005999)

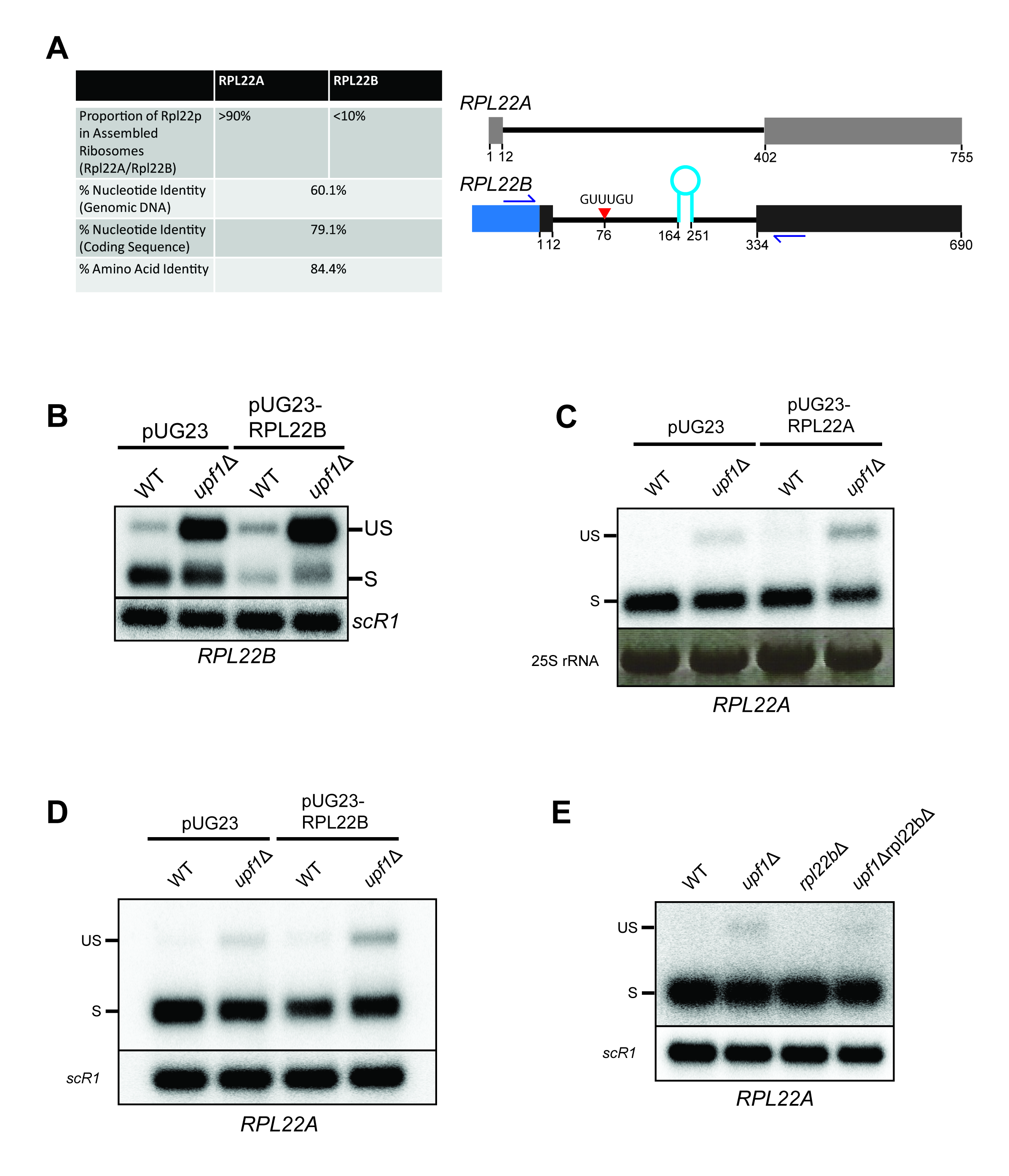

Supplement: S1 Fig — A. Left: Comparative table for RPL22A and RPL22B. Assembled ribosome proportions and percent nucleotide and amino acid identities are provided. Right: Scale diagrams comparing RPL22A (upper) and RPL22B (lower) pre-mRNAs, with the exonic sequences in grey and the introns represented by black lines. The nucleotide positions are labeled with respect to the first nucleotide of the coding sequences. The 5’UTR (blue), alternative 5’-splice site, regulatory element, and RT-PCR primer positions are shown for RPL22B. B. Northern blot detecting the splicing of RPL22B pre-mRNA in wild-type and upf1Δ mutant strains carrying the empty pUG23 vector or the RPL22B overexpression plasmid. Labeled bands show the unspliced (US) and spliced (S) species. Transcripts were detected using an RPL22B 5’UTR riboprobe. SCR1 was used as a loading control. C. Northern blot detecting the splicing of RPL22A pre-mRNA in wild-type and upf1Δ mutant strains carrying the empty pUG23 vector or the RPL22A overexpression plasmid. Transcripts are labeled similarly to panel A and were detected using an RPL22A 3’UTR riboprobe. SCR1 was used as a loading control. D. Northern blot detecting the splicing of RPL22A pre-mRNA in wild-type and upf1Δ mutant strains carrying the empty pUG23 vector or the RPL22B overexpression plasmid. Detection methods and labels are similar to panel C. E. Northern blot detecting the splicing of RPL22A pre-mRNA in wild-type, upf1Δ, rpl22bΔ, and upf1Δrpl22bΔ mutant strains. Detection methods and labels are similar to panel C. (TIF) [file pgen.1005999.s001.tif]

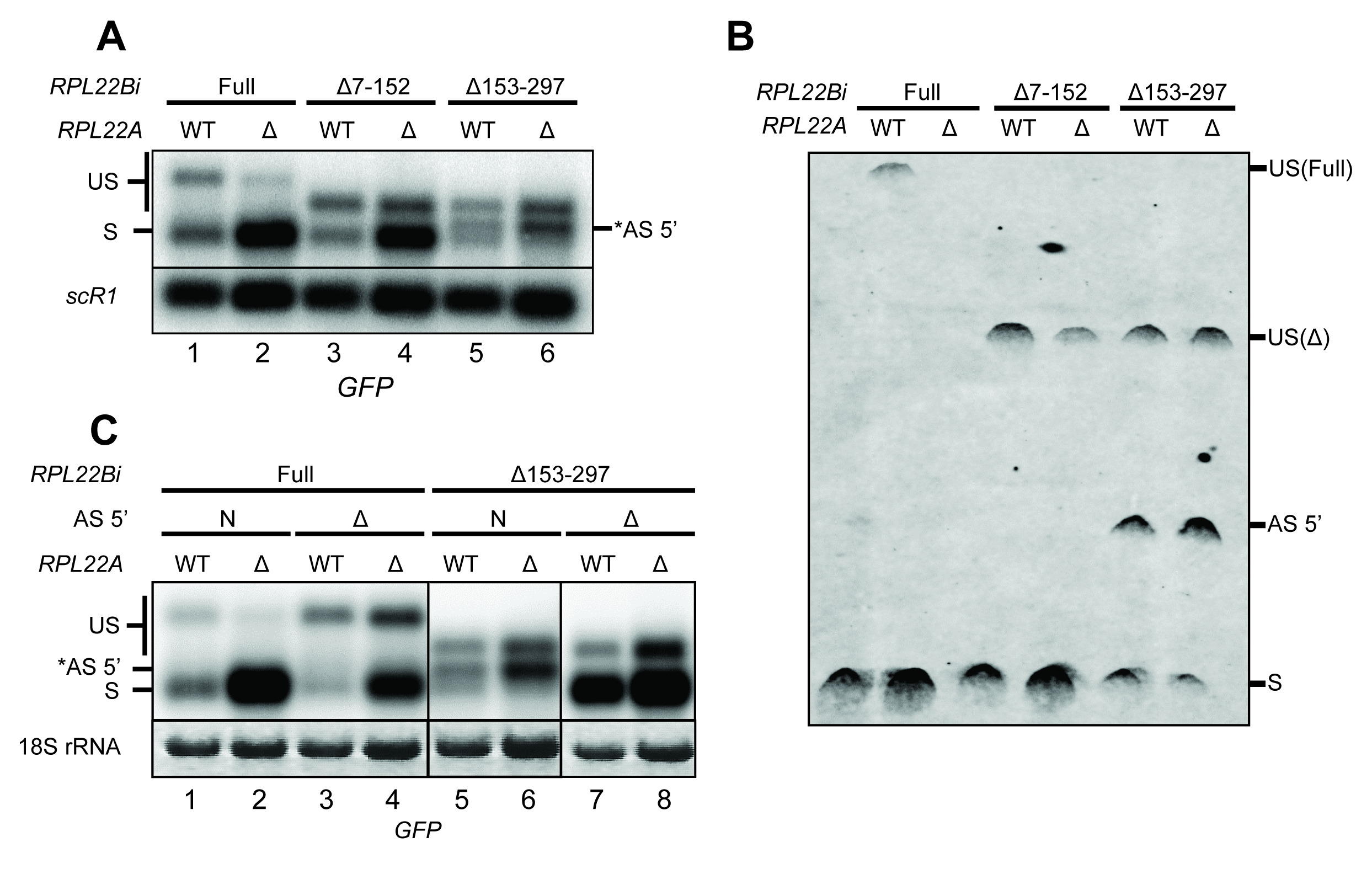

Supplement: S2 Fig — A. Northern blot detecting splicing of the RPL22B intron reporter transcript expressed from constructs with the full intron or deletion of intronic bases 7 though 152 (Δ7–152) or 153 through 297 (Δ153–297) in strains with or without the chromosomal deletion of RPL22A. Labeled bands show the unspliced (US), alternatively-spliced (*AS 5’), and spliced (S) species. Transcripts were detected using a GFP ORF riboprobe. SCR1 was used as a loading control. B. RT-PCR analysis of the RPL22B intron reporter transcript expressed from constructs with full intron, Δ7–152, or Δ153–297 in strains with or without the chromosomal deletion of RPL22A. C. Northern blot detecting splicing of the RPL22B intron reporter transcript expressed from constructs with the full intron, Δ7–152, or Δ153–297 in strains with or without the chromosomal deletion of RPL22A and with or without the deletion of the RPL22B alternative 5’ splice site. Bands are labeled similarly to panel B. Ethidium bromide-stained 18S rRNA is shown as a loading control. (TIF) [file pgen.1005999.s002.tif]

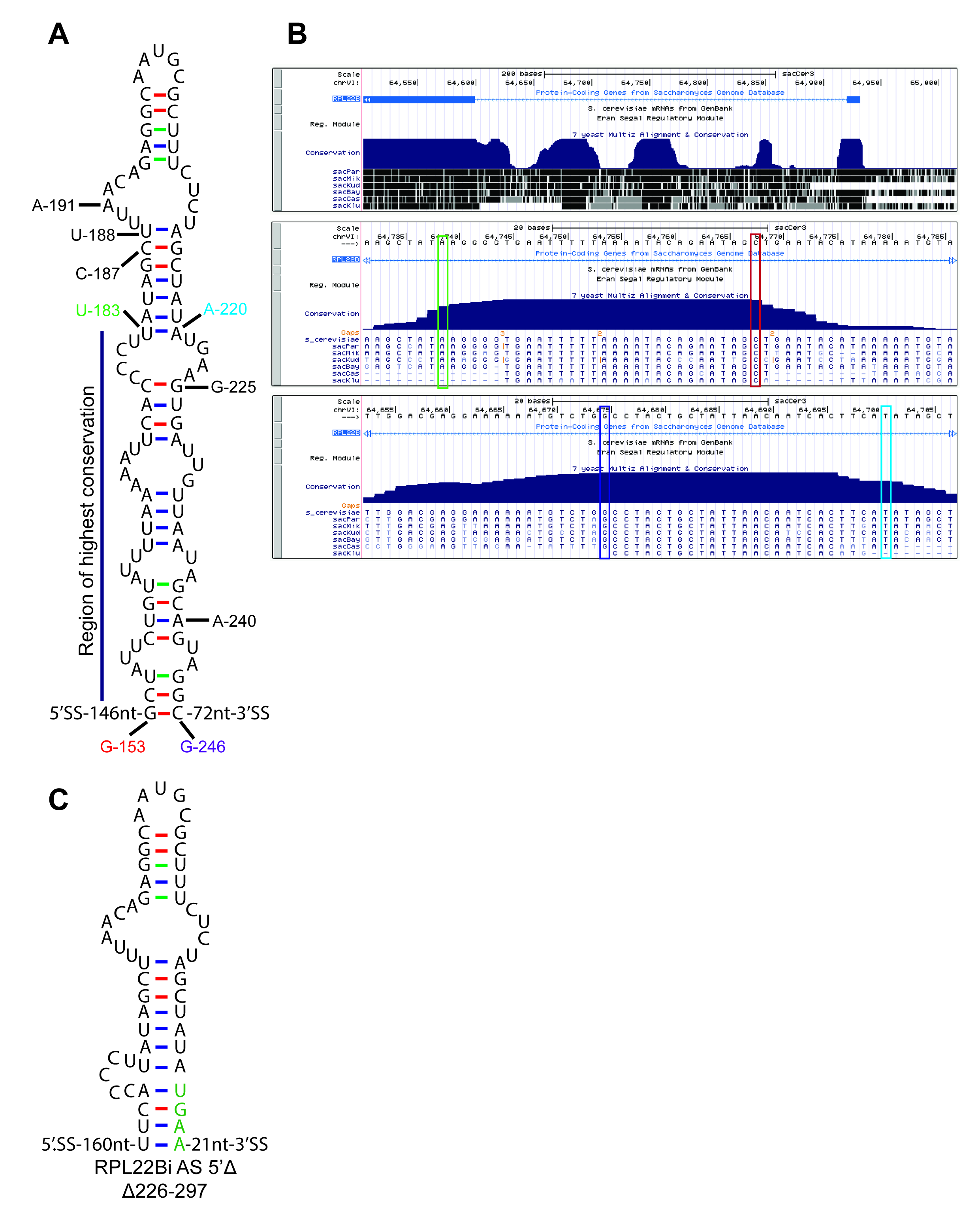

Supplement: S3 Fig — A. The full regulatory element as predicted by Mfold with key nucleotides labeled for reference. Highly conserved nucleotides include both the upstream and downstream nucleotides and are denoted with the black bar. Nucleotides G-153, U-183, A-220 and G-246 have been labeled in colors corresponding to the boxes in the accompanying conservation plots in panel B. B. UCSC genome browser snapshots showing strong sequence conservation of intronic nucleotides forming the lower regions of the regulatory element among yeast. Top panel: Snapshot of the entire intron. Center panel: Snapshot showing conservation of the upstream nucleotides constituting the bottom half of the stem loop, corresponding to intronic nucleotides 153 through 183. Bottom panel: Snapshot showing conservation of the downstream nucleotides constituting the bottom half of the stem loop, corresponding to intronic nucleotides 220 through 246. Note that these plots provide the sequence of the Watson strand while RPL22B is encoded on the Crick strand. C. Structure of the regulatory element as predicted by Mfold when intronic bases 226 through 297 are removed. The nucleotides in green have been determined to promote the regulation of splicing (see text and S5 Fig). (TIF) [file pgen.1005999.s003.tif]

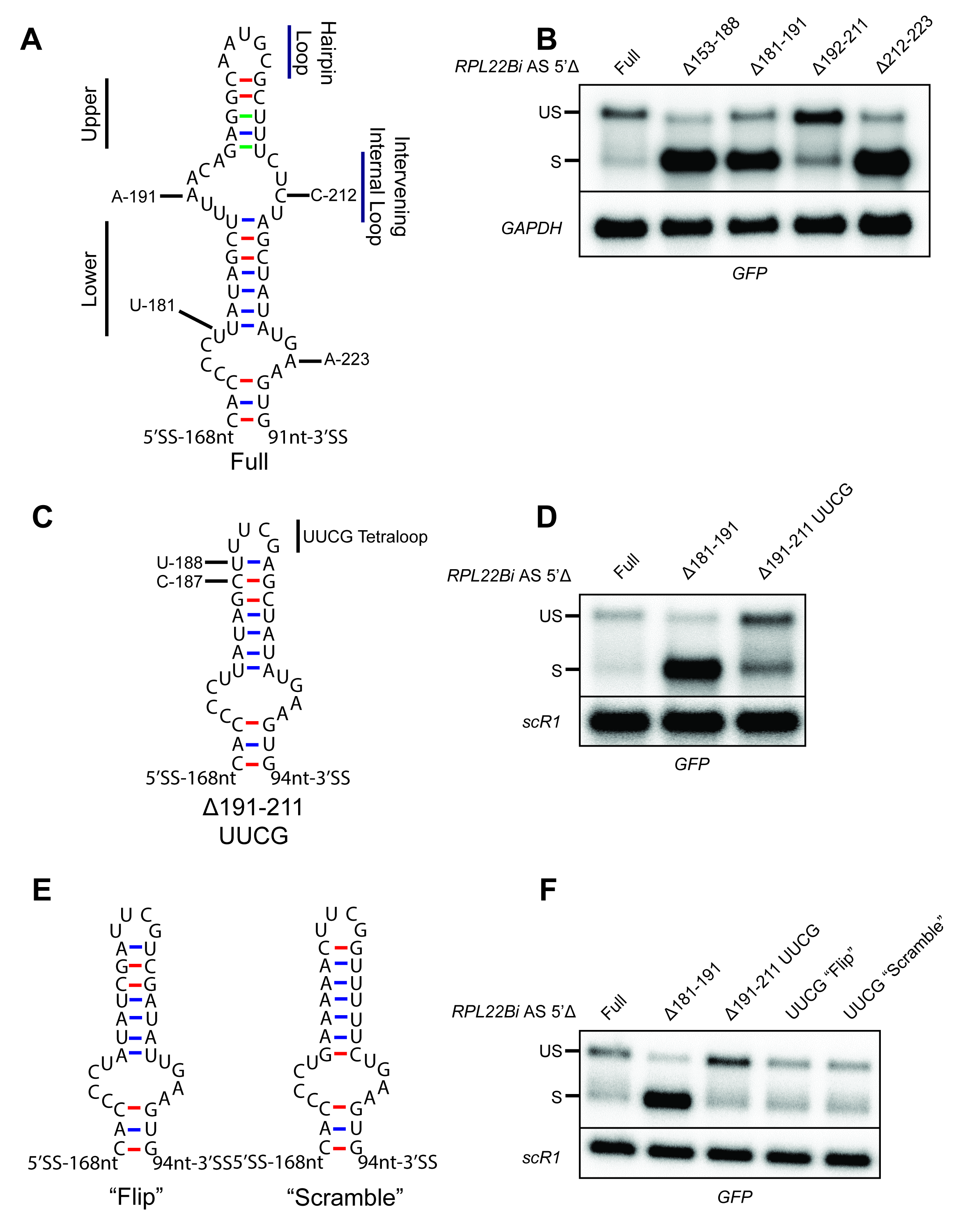

Supplement: S4 Fig — A. Predicted Mfold structure of the distal end of the Full regulatory element showing the upper and lower distal stems, the hairpin loop, and the intervening RNA loop between the two distal stems. B. Northern blot detecting the splicing of the RPL22B intron reporter transcript expressed from constructs with full intron, Δ153–188, or deletion of intronic bases 181 through 191 (Δ181–191) or 212 through 223 (Δ212–223) in wild-type cells. Bands are labeled similarly to panels D-E. Transcripts were detecting using a GFP ORF riboprobe. GAPDH was used as a loading control. C. Predicted Mfold structure of the distal end of the modified construct Δ191–211 UUCG showing the ultrastable tetraloop. D. Northern blot detecting the splicing of the RPL22B intron reporter transcript expressed from constructs with full intron, Δ181–191, and the Δ191–211 UUCG construct in wild-type cells. Bands are labeled similarly to panels D-F. SCR1 was used as a loading control. E. Predicted Mfold structures of the “Flip” and “Scramble” constructs, respectively showing the inversion and reordering of the nucleotides of the lower distal stem. F. Northern blot detecting the splicing of the RPL22B intron reporter transcript expressed from constructs with full intron, Δ181–191, Δ191–211 UUCG, or Δ191–211 UUCG with the lower distal stem bases flipped (Δ191–211 UUCG “Flip”) or scrambled (Δ191–2211 UUCG “Scramble”) in wild-type cells. Bands are labeled similarly to panels B and D. SCR1 was used as a loading control. (TIF) [file pgen.1005999.s004.tif]

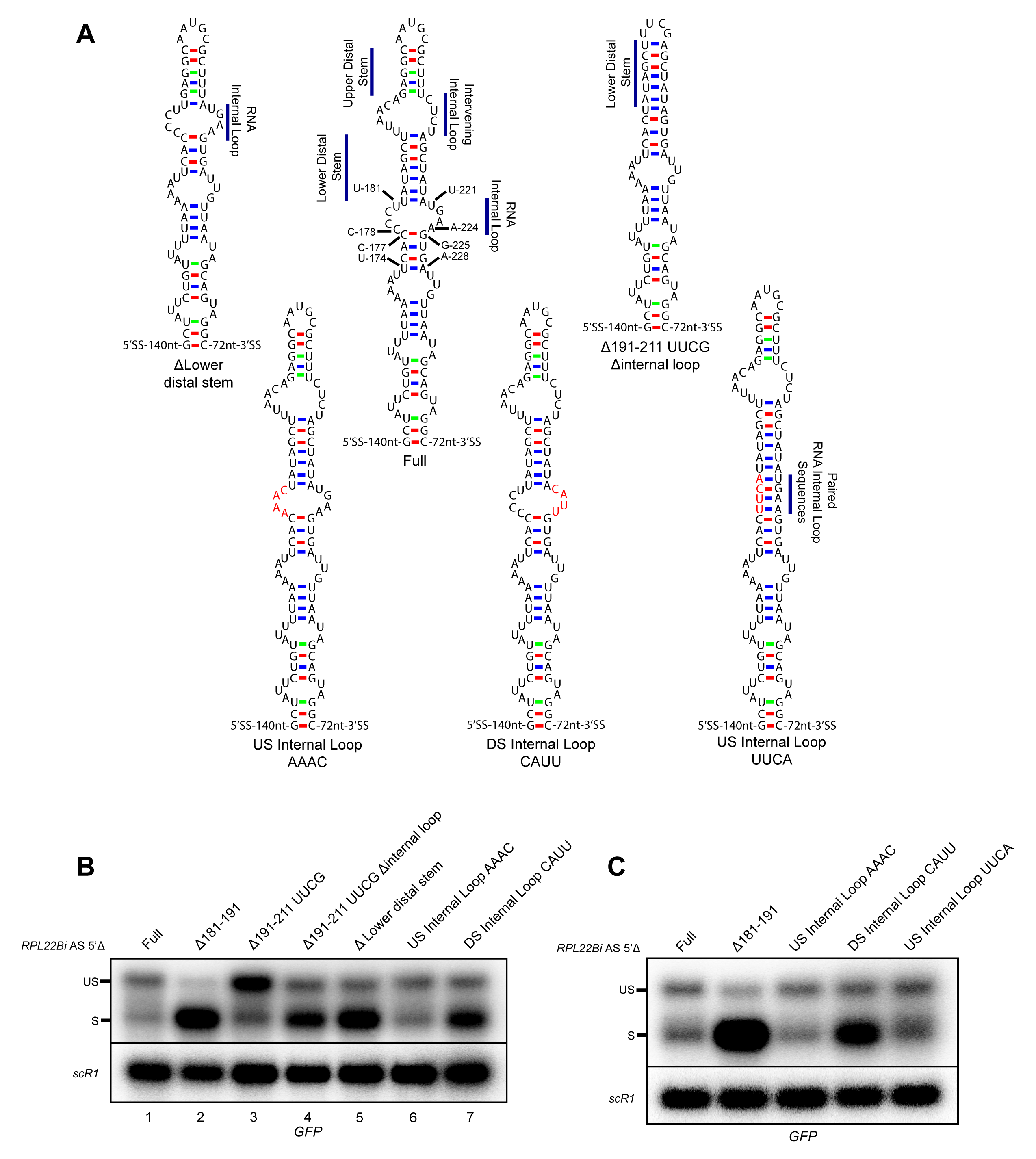

Supplement: S5 Fig — A. Predicted Mfold structures of constructs tested in panels B and C. Nucleotides of interest are colored red. B. Northern blot analysis of RPL22B intron reporter transcript expressed from constructs with full intron, Δ181–191, Δ191–211 UUCG, Δ191–211 UUCG with the deletion of the RNA internal loop (Δ191–211 UUCG Δinternal loop), deletion of the lower distal stem and intervening internal loop (Δlower distal stem), mutation of the upstream RNA internal loop sequence from CCCU to AAAC (US Internal Loop AAAC), or mutation of the downstream RNA internal loop sequence from UGAA to CAUU (DS Internal Loop CAUU) in wild-type cells. Labeled bands indicate the unspliced (US) and spliced (S) species. Bands were detected using a GFP ORF riboprobe. SCR1 was used as a loading control. C. Northern blot analysis of RPL22B intron reporter transcript expressed from constructs with full intron, Δ181–191, US Internal Loop AAAC, DS Internal Loop CAUU, or mutation of the upstream RNA internal loop sequence from CCCU to UUCA (US Internal Loop UUCA). Bands are labeled similarly to panel A. SCR1 was used as a loading control. (TIF) [file pgen.1005999.s005.tif]

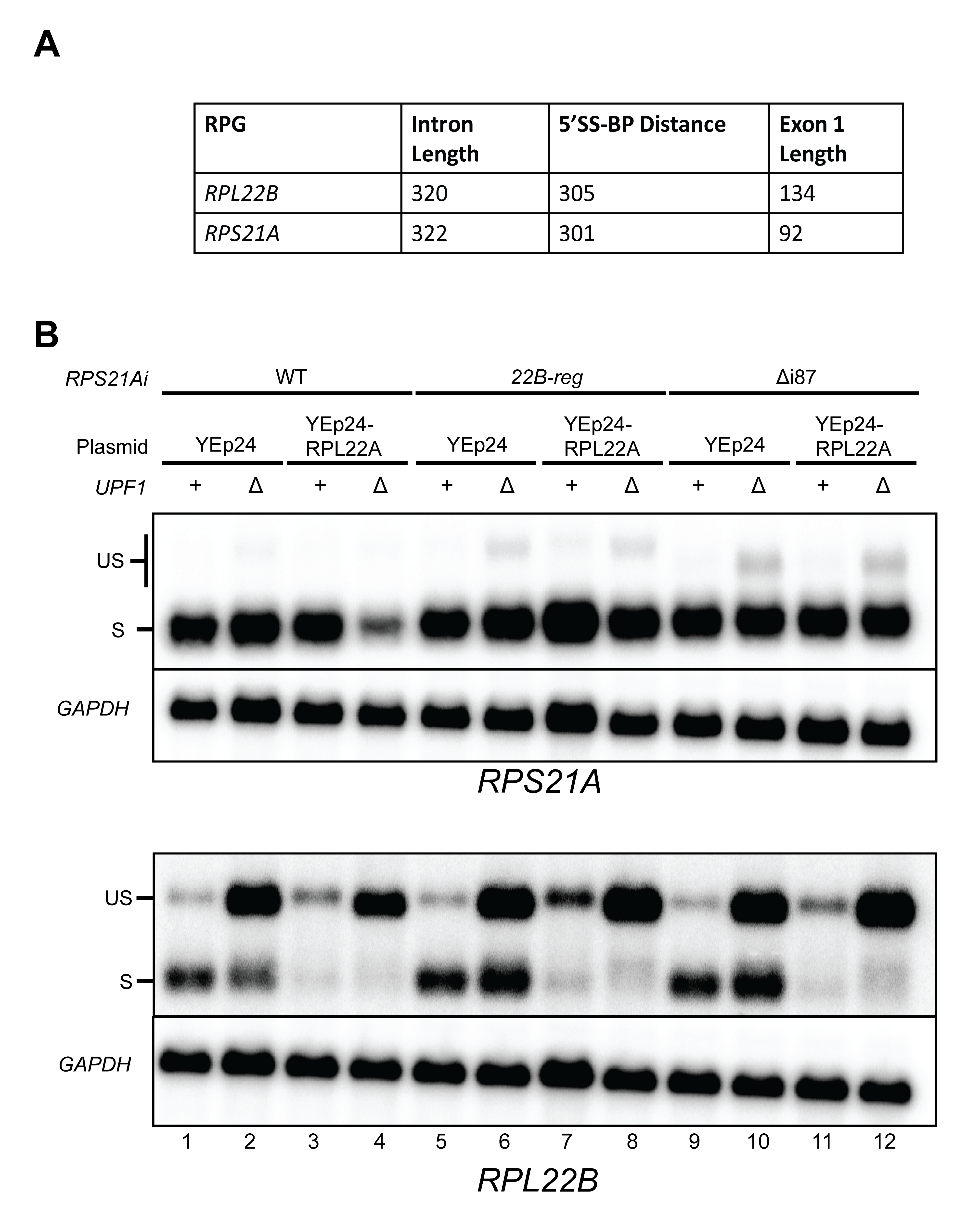

Supplement: S6 Fig — A. Comparison of key intronic properties of RPL22B and RPS21A. B. Northern blots detecting the splicing patterns of RPS21A (upper panel) and RPL22B (lower panel) in strains with the empty YEp24 vector or the RPL22A overexpression plasmid. The strains carry either the wild-type RPS21A intron or mutated versions of the intron harboring either the RPL22B regulatory element (22B-reg) or the an 87 nucleotide intronic deletion (Δi87). GAPDH was used as a loading control. See S1 Text for additional details. (TIF) [file pgen.1005999.s006.tif]

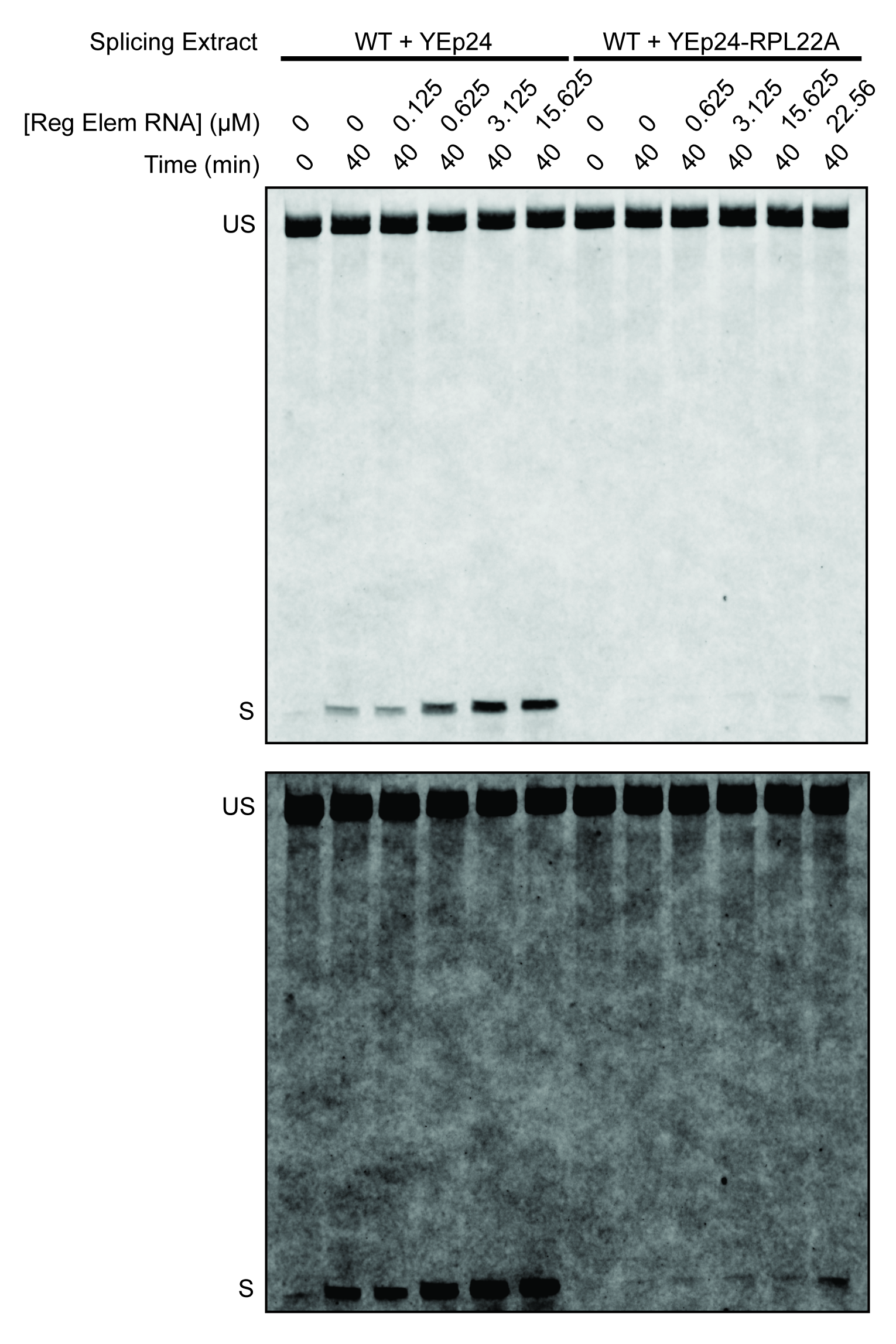

Supplement: S7 Fig — These are non-attenuated images of the gels that are presented in truncated form in Fig 4E. (TIF) [file pgen.1005999.s007.tif]

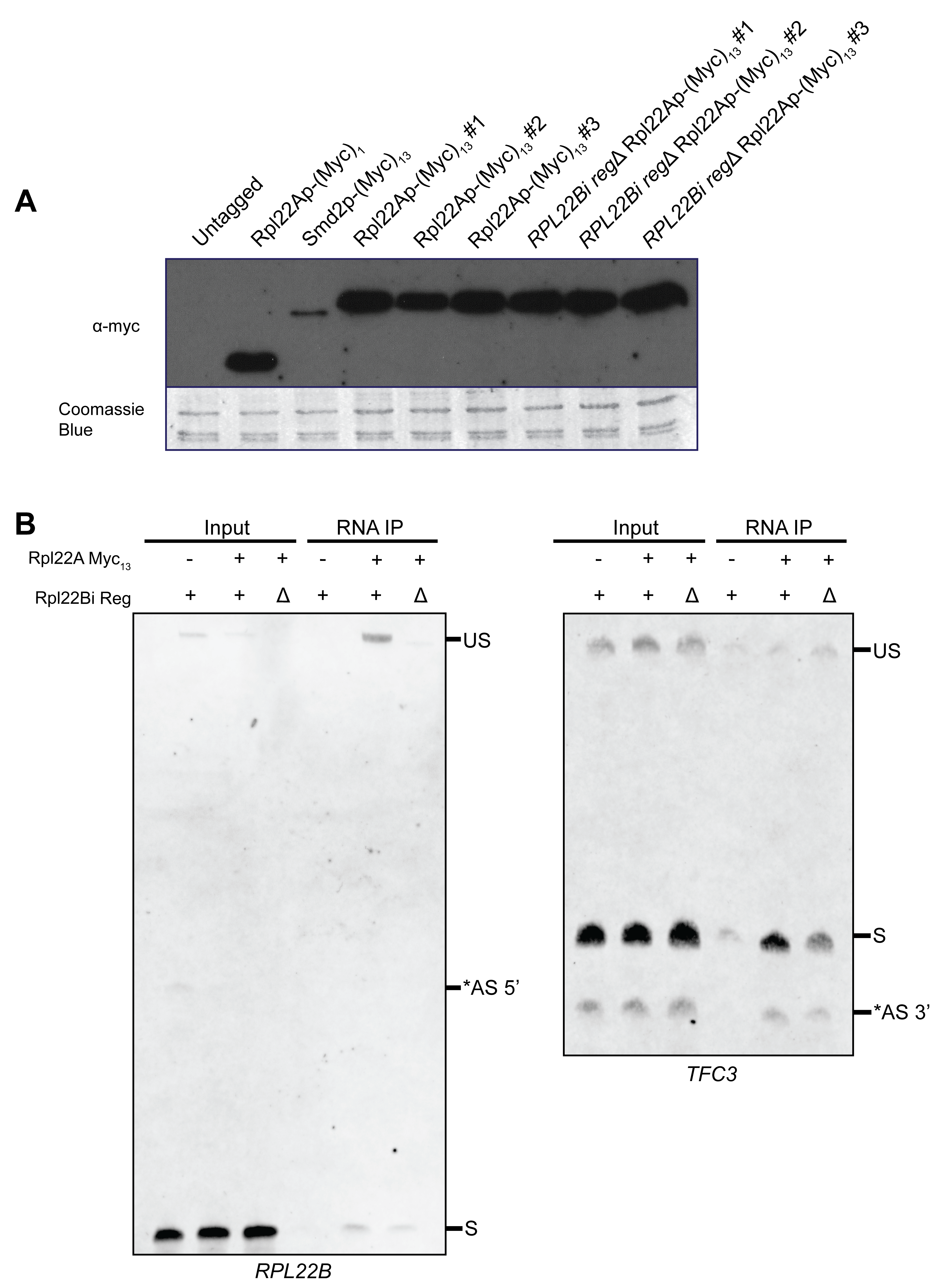

Supplement: S8 Fig — A. Western blot confirmation of the Myc epitope tag on Rpl22Ap. The untagged strain was used as a negative control. Strains expressing Rpl22Ap with a single epitope tag and a Myc-tagged Smd2 protein were used as positive controls. Three separate clones of strains expressing 13-Myc tagged Rpl22Ap were each tested in WT background and the RPL22B intron regulatory element deletion background (RPL22Bi regΔ). The Myc epitope tag was detected using an anti-Myc primary antibody. Coomassie blue stained proteins are shown as a loading control. B. Full unattenuated gel images that were presented in truncated form in Fig 3D. (TIF) [file pgen.1005999.s008.tif]

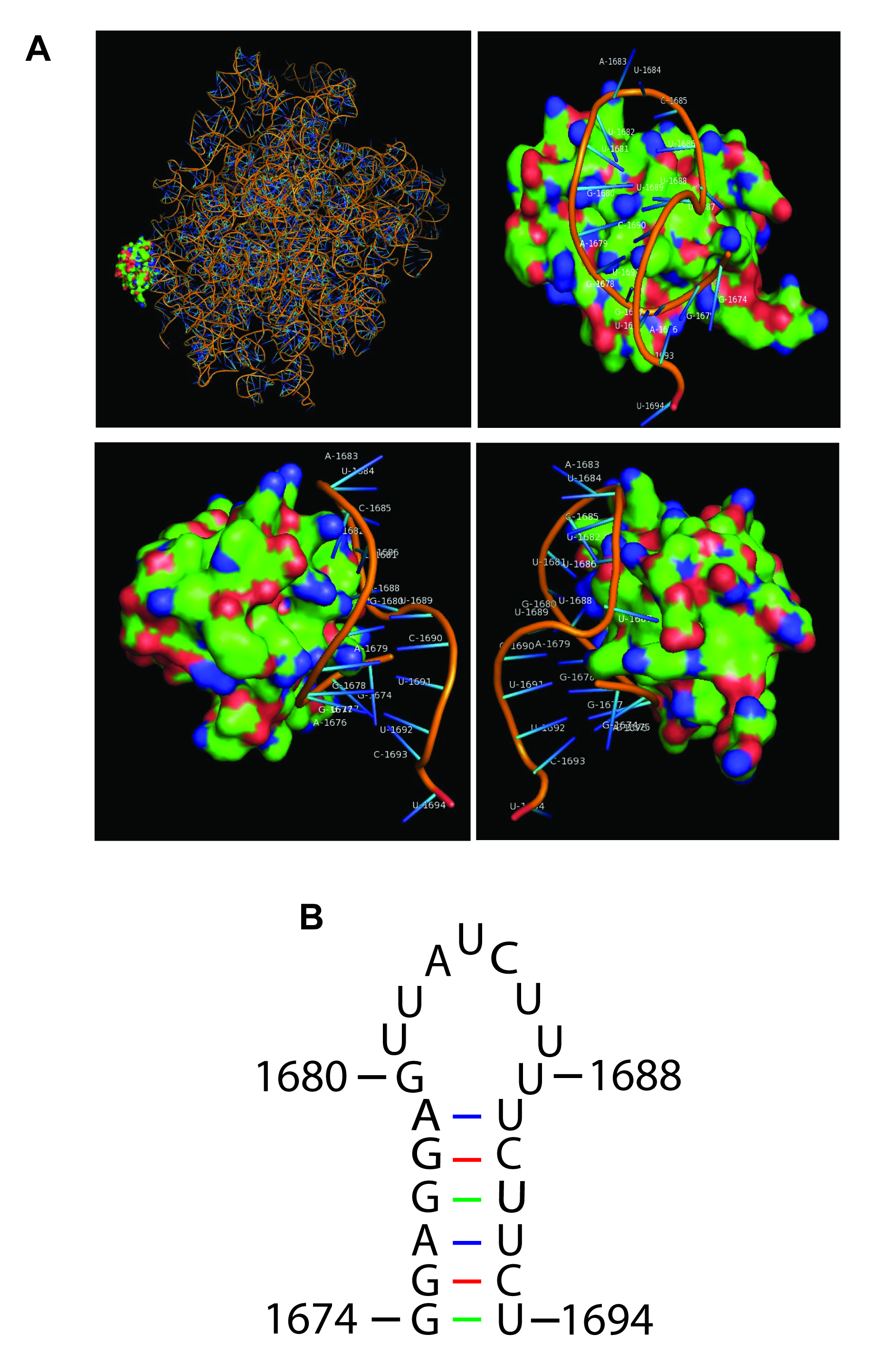

Supplement: S9 Fig — A. PyMol 3D views of the yeast Rpl22 protein and the proximal rRNA sequence. Clockwise from upper left: Rpl22p in the context of all large subunit rRNA, frontal view of Rpl22 and proximal rRNA secondary structure, left side view, and right side view. Protein and RNA structures are from [34]. B. 2D representation of the rRNA secondary structure from [35]. (TIF) [file pgen.1005999.s009.tif]

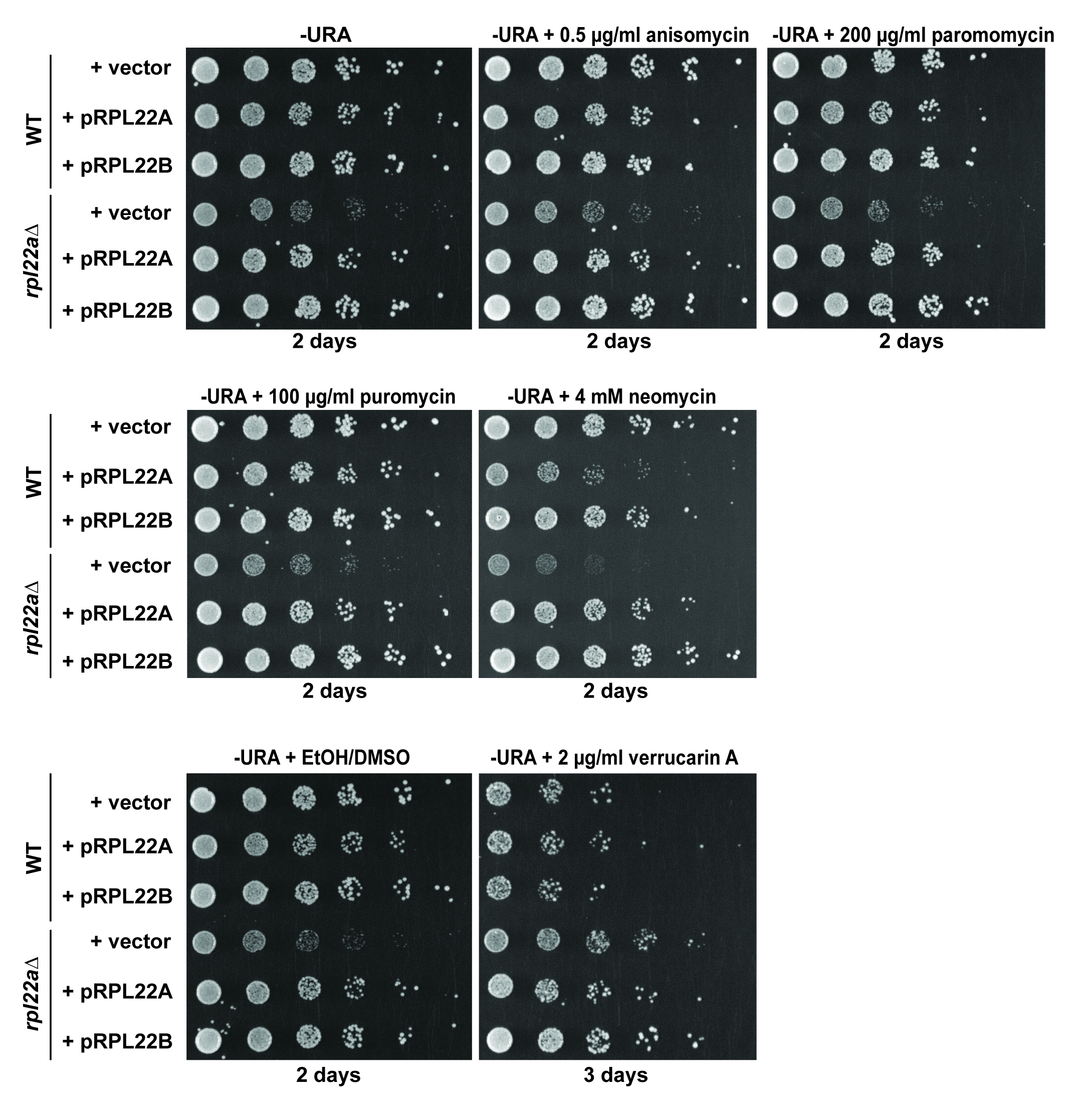

Supplement: S10 Fig — Growth of WT and rpl22aΔ strains carrying an empty YEp24 vector or RPL22 paralog overexpression plasmids in various ribosome-inhibiting antibiotics. Following 5-fold spot dilution, plates were incubated at 30°C for the number of days indicated. (TIF) [file pgen.1005999.s010.tif]

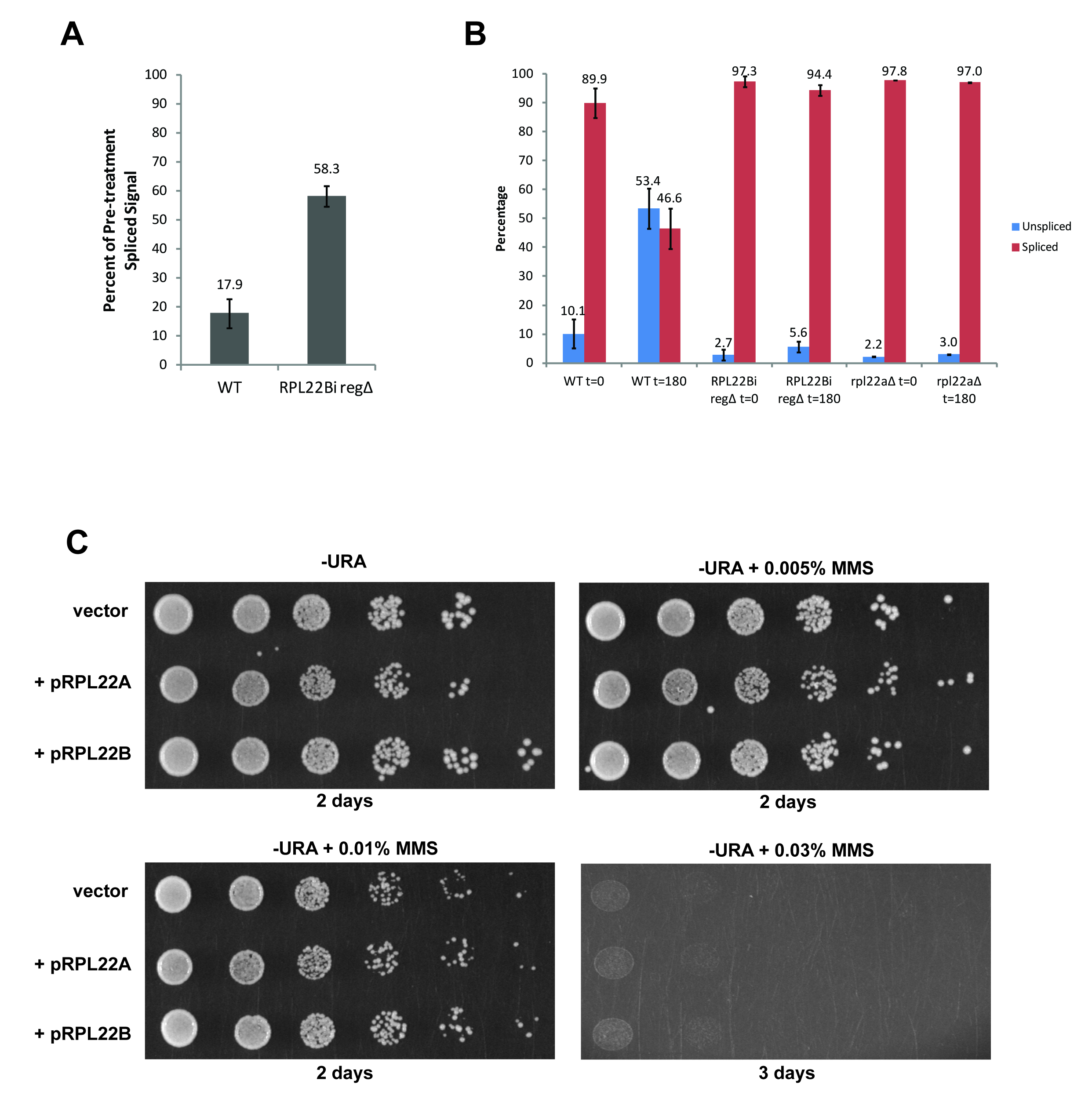

Supplement: S11 Fig — A. Quantification of spliced RPL22B transcript in WT and RPL22Bi regΔ strains after three hours of treatment with 0.05% MMS reported as a percentage of spliced transcript that was present prior to treatment (t = 0). The values shown are mean ± 1 standard deviation measured from Northern blot experiments consisting of three independent biological replicates for each strain. B. Quantification of unspliced and spliced RPL22B transcripts in wild-type, RPL22Bi regΔ, and rpl22aΔ strains at steady state and following three hours of treatment with 0.05% MMS, given as percentage of the total transcript. The values shown are mean ± 1 standard deviation measured from Northern blot experiments consisting of three independent biological replicates for each strain. C. Wild-type cells carrying an empty YEp24 vector or plasmids overexpressing the intronless cDNA of RPL22A and RPL22B were tested for growth in 0%, 0.005%, 0.01% and 0.03% MMS. Following 5-fold spot dilution, plates were incubated at 30°C for the number of days indicated. (TIF) [file pgen.1005999.s011.tif]

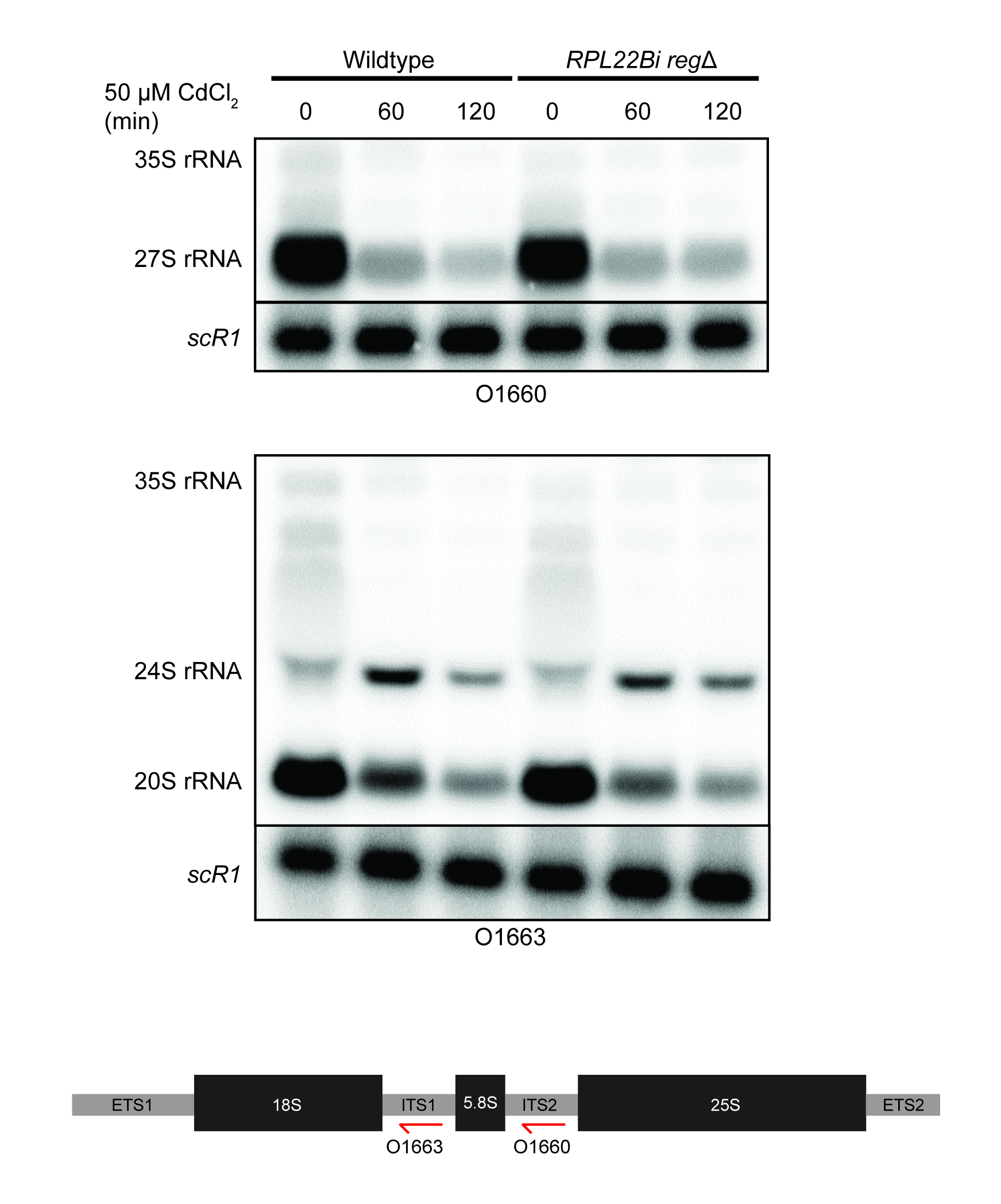

Supplement: S12 Fig — Northern blots detecting the 35S rRNA and other rRNA processing intermediates. Cells were grown in YPD containing 50 μM CdCl2 for 120 minutes. The 35S rRNA and processing intermediates were detected using oligoprobes complementary to transcripts arising from the RDN1 locus as shown in the lower diagram. Sequences for O1660 and O1663 were obtained from Lindahl et al. [59]. SCR1 was used as a loading control. (TIF) [file pgen.1005999.s012.tif]

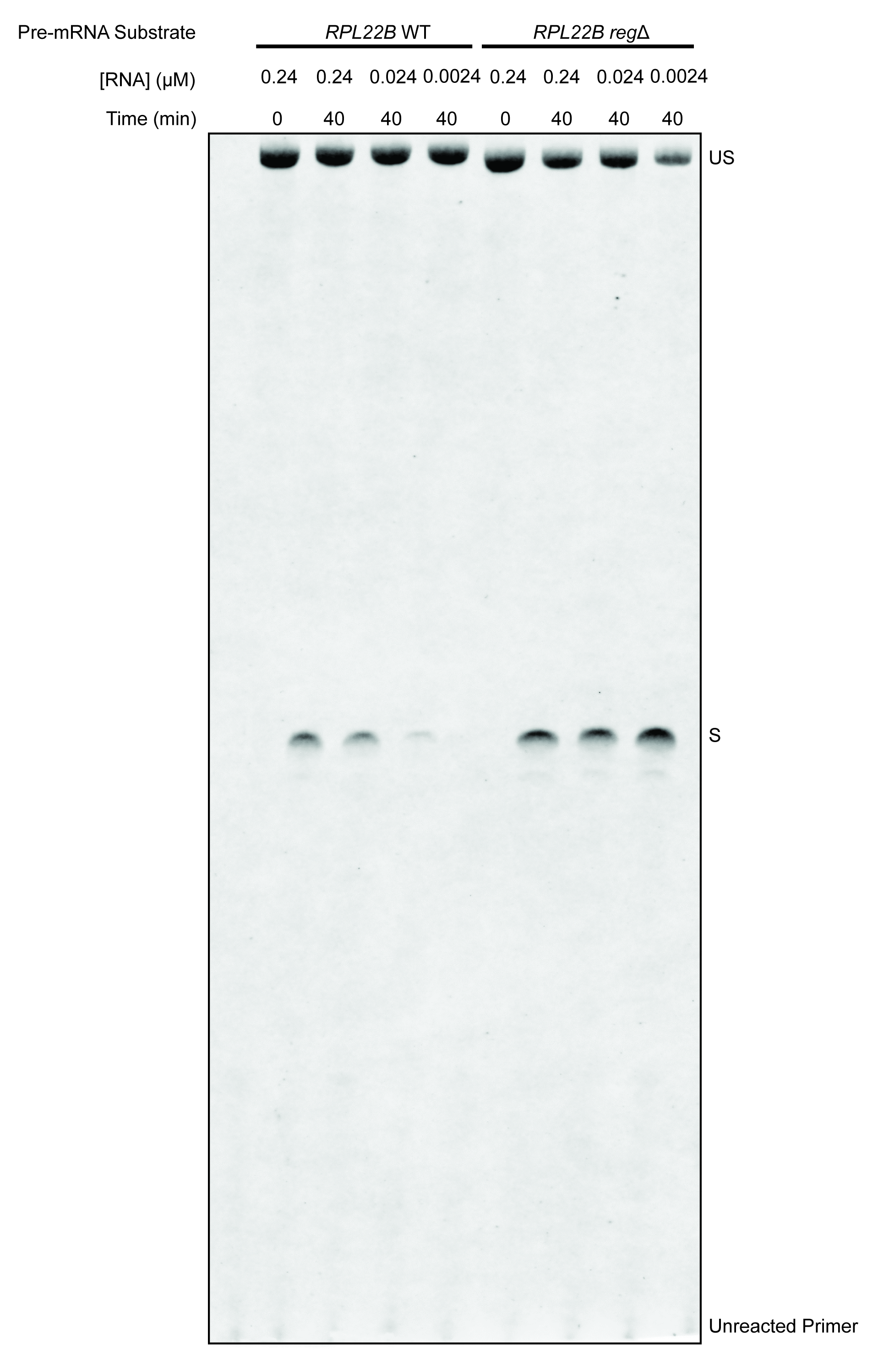

Supplement: S13 Fig — This is a non-attenuated image of the gel that is presented in truncated form in Fig 4C. (TIF) [file pgen.1005999.s013.tif]

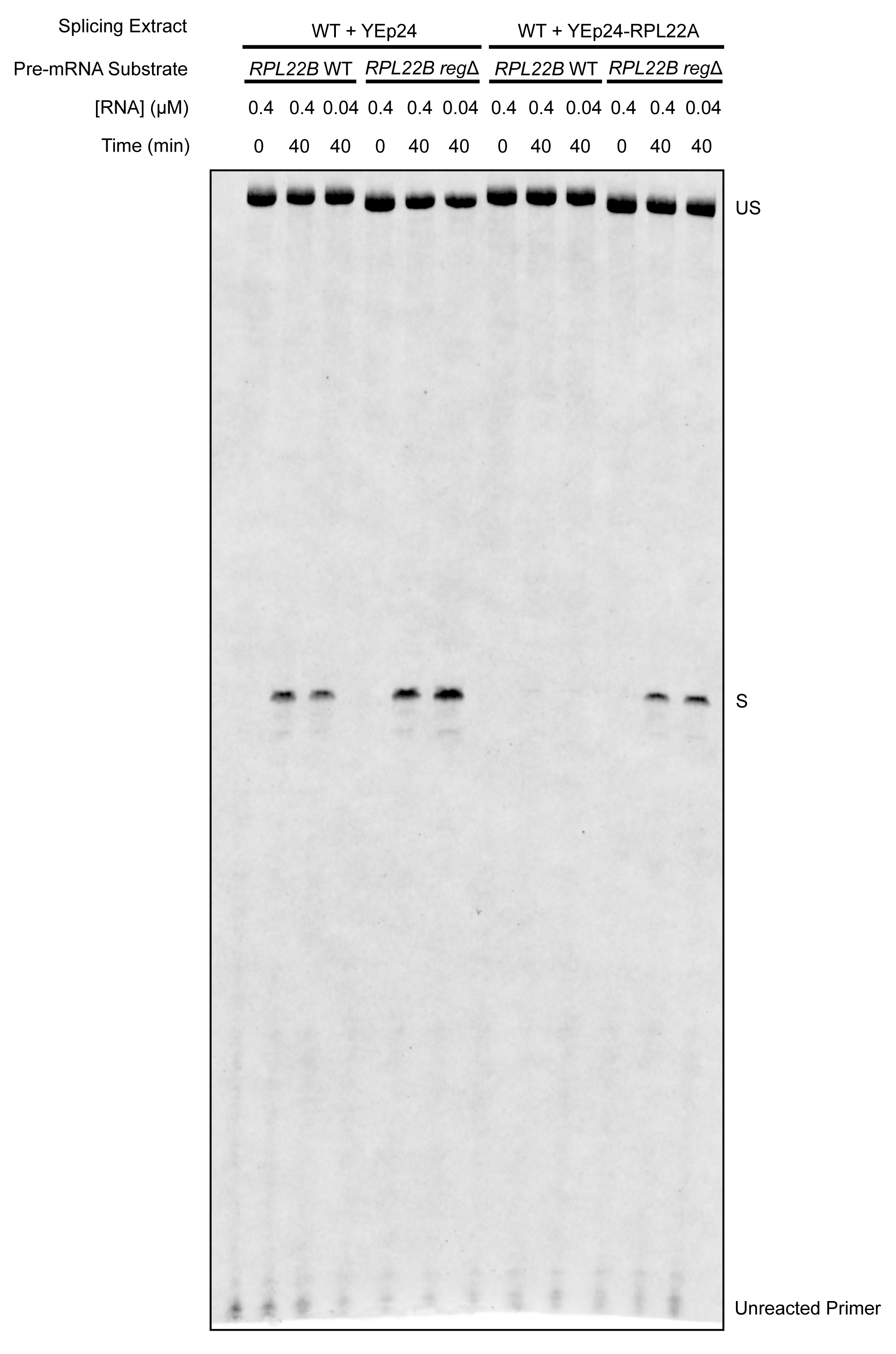

Supplement: S14 Fig — This is a non-attenuated image of the gel that is presented in truncated form in Fig 4D. (TIF) [file pgen.1005999.s014.tif]
