# Supplementary material for: Splicing-Mediated Autoregulation Modulates Rpl22p Expression in Saccharomyces cerevisiae
Source: PLoS Genet. 2016 Apr 20;12(4):e1005999. doi: 10.1371/journal.pgen.1005999 (PMC4838235; doi:10.1371/journal.pgen.1005999)
Supplement: S1 Text — (DOCX) [file pgen.1005999.s015.docx]

**Binary search approach**

We began the search for the *RPL22B* intronic regulatory element by making systematic bifurcating deletion of the GFP reporter transcript as outlined in Fig 3A. Our initial experimental system utilized the *rpl22a*Δ strain as a control for uninhibited splicing based on the presumption that the removal of the regulatory element from the intron would phenocopy the loss of splicing inhibition for *RPL22B* that is observed in the absence of *RPL22A* (Fig 1C). Indeed, the splicing of the reporter transcript containing the full intron was greatly enhanced in the *rpl22a*Δ strain compared to wildtype (S2A Fig, lanes 1 and 2). The first set of deletions removed either intronic nucleotides 7 through 152 (Δ7-152) or nucleotides 153 through 297 (Δ153-297) from the reporter (Fig 3A). The former deletion had no impact on reporter splicing efficiency in the presence of *RPL22A*, indicating that this region did not harbor the regulatory sequence (S2A Fig, lanes 3 and 4). Surprisingly, the Δ153-297 deletion also did not enhance the splicing of the reporter (S2A Fig, lane 5). Furthermore, splicing efficiency was not enhanced in the *rpl22a*Δ strain for the Δ153-297 construct (S2A Fig, lane 6), suggesting that the assay was not functioning as expected in this particular deletion construct. However, an additional splice product running slightly higher than the normal spliced product was also detected in this deletion. We therefore re-analyzed the splicing patterns by RT-PCR and we found that the deletion of nucleotides 153 through 297 promoted the use of the alternative 5’ splice site (AS 5’) previously described for *RPL22B* (S2B Fig), which in turn interfered with the assay. A band representing the alternatively spliced species was clearly visible in the Δ153-297 strains in addition to the unspliced and normal splice products (S2B Fig). The reason for this change in preferred splice site selection in this deletion construct is not clear, but is possibly related to the impact of physical spacing between the splice sites and the branch point [1-3].

Because the presence of the AS 5’ within the *RPL22B* intron obstructed our ability to analyze sequences required for *RPL22B* splicing regulation, we removed the AS 5’ through site-directed mutagenesis for the remainder of our search for the regulatory element. The deletion of the AS 5’ restored the functionality of the assay, revealing a substantial increase in splicing efficiency in the Δ153-297 construct when compared to the full-length construct even in the presence of *RPL22A* (S2C Fig, lanes 3 and 7). Thus, the regulatory element resides within the region spanning nucleotides 153 through 297 of the *RPL22B* intron. Interestingly, deletion of the AS 5’ also appeared to reduce overall splicing efficiency at the annotated 5’ splice site in the full-length transcript (S2C Fig, lanes 2 and 4), hinting that the presence of dual 5’ splice sites may increase spliceosome assembly efficiency *in vivo* for *RPL22B*.

The second set of intronic truncations consisted of deletions of nucleotides 153 through 225 and 226 through 297. The Δ153-225 deletion eliminated the inhibition of splicing while the Δ226-297 deletion maintained it (Fig 3A, top right panel), thus narrowing the regulatory element to the span of nucleotides 153 through 225. Finally, the third series of truncations deleted nucleotides 153 through 188 and 189 through 225. Splicing inhibition was eliminated in both cases (Fig 3A, bottom right panel), suggesting that the regulatory element consists of nucleotides from both of these regions. We were therefore unable to further pinpoint the regulatory element using the binary search approach and were required to conduct more specific targeted mutations of this region.

**The intronic segment containing the regulatory element is predicted to form a structured stem loop**

Upon identifyi­ng the general location of the regulatory element as within the region bordered by intronic nucleotides 153 through 225, we examined the predicted structure of the *RPL22B* intron as determined by the Mfold web server. We found that this span of intronic nucleotides has the potential to fold into a stem loop that includes nucleotides 153 through 246 and that might constitute an RNA secondary structure required for splicing inhibition (S3A Fig). Intriguingly, the nucleotides constituting approximately the lower half of this stem loop are highly conserved amongst related yeast species (S3A Fig and S3B Fig). This contrasts with the general lack of conservation the intronic sequence, suggesting that these sequences may be functionally relevant and perhaps serve a role in the inhibition of splicing. However, our deletion construct Δ226-297 maintained inhibition of splicing, even though it is predicted to remove most of the lower conserved regions from the secondary structure and leave only the upper portions structurally intact (Fig 3A and S3C Fig). This suggests that the majority of conserved nucleotides are not necessary for splicing inhibition, although we cannot rule out the possibility that they may indirectly contribute to the overall structure by enhancing the exposure of the remainder of the stem loop or contribute to the folding of the tertiary structure.

**Secondary structure, not sequence specificity, of the lower distal stem is required for inhibition of splicing**

Our previous experiments demonstrated the necessity of the approximate upper (distal) half of the stem loop of this secondary structure for the bestowal of splicing inhibition. This region consists of a lower stem, an intervening internal loop, and an upper stem that is capped by an AAUGC hairpin pentaloop (S4A Fig). All of these features are potential candidates for RNA-protein interactions [4, 5]. To further dissect these components, we disrupted this region by creating three additional independent deletion constructs. The first, Δ181-191, removes the 5’ nucleotides from the lower stem and was predicted by Mfold to dramatically reshape the intron such that the putative regulatory element no longer forms. The second construct, Δ192-211, removes the upper stem loop and intervening internal loop while maintaining the lower stem, closing it with a UUACU pentaloop and also preserving the overall secondary structure of the entire intron. The third construct, Δ212-223, removes the 3’ nucleotides from the lower stem and is likewise expected to completely alter the intronic secondary structure. Not surprisingly, constructs Δ181-191 and Δ212-223 did not exhibit splicing regulation by Rpl22p (S4B Fig). However, splicing regulation by Rpl22p was maintained in construct Δ192-211, suggesting that the upper stem and hairpin loop are not required for recognition by Rpl22p and subsequent inhibitory effects, while the lower stem is essential (S4B Fig).

We next were interested in determining what facets of the lower stem region contribute to splicing regulation. First, we truncated the full structure by removing the upper stem and its hairpin loop (both of which were demonstrated as nonessential in the previous experiment) and closing the lower stem with a UUCG tetraloop, creating the construct Δ191-211 UUCG (S4C Fig). This hairpin sequence is a member of the UNCG family of thermodynamically stable loop sequences [6], enabling further manipulations of the remaining stem nucleotides with minimal risk of dramatically altering the structure. Consistent with our previous experiments, the replacement of the upper distal stem loop with the UUCG tetraloop sequence retained splicing regulation (S4D Fig). To unambiguously determine whether sequence identity of the lower stem is required to maintain the regulatory mechanism, we generated two additional constructs in which the identities of the stem nucleotides were altered but secondary structure was maintained. In the first construct (“Flip”), we reversed the positions of the upstream 5’ and downstream 3’ stem sequences (S4E Fig, left). In the second construct (“Scramble”), we shifted the positions of the stem base pairs while maintaining the identities of the pairs themselves – that is, we maintained the two G-C pairs and five A-U pairs (S4E Fig, right). Neither mutation enhanced the splicing of the reporter (S4G Fig), supporting the argument that the structure and not the sequence identity of this region is necessary for the inhibition of splicing. However, although our previous constructs that disrupted this stem (Δ181-191 and Δ212-223) demonstrated a loss of regulation by Rpl22p, we could not rule out that this was a result of the dramatic restructuring of the intron that may also have affected other critical areas of the regulatory element. To account for this possibility, we deleted this lower stem from the full wild-type structure, leaving the remainder of the regulatory element and the rest of the intron intact (S5A Fig, Δlower distal stem structure). This mutation resulted in a loss of splicing regulation (S5B Fig, lane 5), confirming our hypothesis that the lower distal stem structure is necessary for this mechanism to function, though it is flexible in its sequence identity.

**The downstream nucleotides of the putative RNA internal loop are likely paired with upstream nucleotides**

Truncations of *RPL22B* intron reporter revealed that the upper distal stem loop (nucleotides 191 through 211), the intervening RNA internal loop, and the nucleotides downstream of position 225 are unnecessary for splicing inhibition (Fig 3A). Furthermore, the deletion of nucleotides 226 through 297 eliminated the participation of nucleotides 153 through 172 in the stem (S3C Fig). The resulting preservation of splicing regulation implies that the intronic nucleotides upstream of G153 are not required for the inhibitory mechanism (Fig 3A). Thirdly, our previous experiments showed that the lower distal stem is required for the inhibition of splicing (S4 Fig and S5 Fig). Therefore, the final remaining component in this region that could additionally contribute to the regulatory element was the putative “RNA internal loop” immediately below the lower distal stem formed by upstream nucleotides 178 through 181 and downstream nucleotides 221 through 224 (S5A Fig, see “Full” structure). We deleted these nucleotides from the simplified UUCG tetraloop reporter (S5A Fig, Δ191-211 UUCG Δinternal loop) which resulted in a notable increase in splicing, albeit not to the extent seen when the entire regulatory element is disrupted (S5B Fig, lanes 2 and 4). This suggests that the putative internal loop is partially involved in the inhibition of splicing. In agreement with this notion, the Δlower distal stem construct retains the RNA internal loop nucleotides and yet loses the ability to inhibit splicing (S5A Fig and S5B Fig), suggesting that this loop or its constituent nucleotides contribute to the regulatory mechanism but alone are insufficient to confer it.

We also investigated whether the sequence identity of this potential internal loop region is crucial for its role as part of the regulatory element. For this experiment we worked from the full intron construct (S5A Fig) and created two new reporters. In the first, the four upstream internal loop nucleotides were mutated from CCCU to AAAC (S5A Fig, “US Internal Loop AAAC”). In the second, the four downstream internal loop nucleotides were mutated from UGAA to CAUU (S5A Fig, “DS Internal Loop CAUU”). In both cases the mutations were predicted to maintain the overall structure of the entire stem loop. Surprisingly, the mutation of the downstream internal loop nucleotides appeared to at least partially mitigate splicing inhibition while mutation of the upstream internal loop had no effect (S5B Fig, lanes 6-7). This result is seemingly contradictory to the behavior of the Δ226-297 construct, which eliminated the downstream internal loop and yet did not disrupt the inhibition of splicing (Fig 3A and S3C Fig). Importantly, the downstream internal loop nucleotides UGAA were retained in that construct but were no longer predicted to constitute a single-stranded internal loop. Instead, they were paired with upstream nucleotides 173 through 176 (UUCA), forming a perfect complement (S3C Fig). Based on this evidence, we hypothesized that the inhibition of splicing depends on the nucleotide identity of the downstream UGAA sequence and that it may actually be a paired structure in its most stable form instead of a single-stranded loop region as predicted by Mfold. To test this idea, we manipulated the full intron reporter construct by mutating the upstream internal loop nucleotides to UUCA, thus establishing a perfect complementarity with the downstream nucleotides in the presumed internal loop. The predicted Mfold structure shows that this mutation closes the internal loop completely by pairing each constituent nucleotide (S5A Fig, “US Internal Loop UUCA”). Closing of the internal loop in this manner did not increase splicing efficiency (S5C Fig), suggesting that the downstream RNA internal loop is actually a paired sequence. Based on our experimental evidence, we specula­­te that in its most stable configuration nucleotides U221 through A224 of the putative RNA internal loop form base pairs with upstream nucleotides U173 through A176. Indeed, constraining this pairing using Mfold results in only one stable predicted structure that very closely resembles the Δ226-297 construct (compare Fig 3B and S3C Fig). Importantly, this experimentally-deduced structure retains the essential lower distal stem.

**The deduced regulatory element is necessary but not sufficient for regulatory activity *in vivo***

The experiments thus far have indicated that the *RPL22B* regulatory element consisting of intronic nucleotides 153 through 239 is necessary for the inhibition of splicing (Fig 3C). To test whether this region is also sufficient for achieve autoregulation, we transposed this span of nucleotides into the intron of *RPS21A*, another RPG whose intron is of similar overall length to that of *RPL22B* and whose branch point is located in a similar relative position relative to the 5’-splice site (S6 Fig). To achieve transposition without impacting overall intron size, we first removed 87 nucleotides from the *RPS21A* intron that occupied the same region of that intron as the regulatory element in the *RPL22B* intron. We then inserted the 87 nucleotides of the *RPL22B* regulatory element into this region using the delitto perfetto technique. The *RPL22A* overexpression plasmid was then introduced into this strain and examined the *RPS21A* transcript in wild-type and NMD mutant background strains to test whether the chimeric intron would experience inhibition of splicing. Although both the deletion of the 87 nucleotides from the *RPS21A* intron as well as the transposition of the *RPL22B* intronic nucleotides impacted the susceptibility of the *RPS21A* pre-mRNA to NMD, it did not render the pre-mRNA sensitive to splicing inhibition by Rpl22p (S6 Fig, compare lanes 1-4 to lanes 5-8). Thus, this span of 87 nucleotides is insufficient to confer inhibition of splicing *in vivo*.

**Supporting References**

1. Cellini A, Felder E, Rossi JJ. Yeast pre-messenger RNA splicing efficiency depends on critical spacing requirements between the branch point and 3' splice site. EMBO J. 1986 May;5(5):1023-30.

2. Crawford DJ, Hoskins AA, Friedman LJ, Gelles J, Moore MJ. Single-molecule colocalization FRET evidence that spliceosome activation precedes stable approach of 5' splice site and branch site. Proc Natl Acad Sci U S A. 2013 Apr 23;110(17):6783-8.

3. Thompson-Jager S, Domdey H. Yeast pre-mRNA splicing requires a minimum distance between the 5' splice site and the internal branch acceptor site. Mol Cell Biol. 1987 Nov;7(11):4010-6.

4. Jones S, Daley DT, Luscombe NM, Berman HM, Thornton JM. Protein-RNA interactions: a structural analysis. Nucleic Acids Res. 2001 Feb 15;29(4):943-54.

5. Nagai K. RNA-protein interactions. Current Opinion in Structural Biology. 1992;2(1):131-7.

6. Molinaro M, Tinoco I. Use of ultra stable UNCG tetraloop hairpins to fold RNA structures: thermodynamic and spectroscopic applications. Nucleic Acids Res. 1995;23(15):3056-63.
